# Homocysteine and Coronary Heart Disease: Meta-analysis of *MTHFR* Case-Control Studies, Avoiding Publication Bias

**DOI:** 10.1371/journal.pmed.1001177

**Published:** 2012-02-21

**Authors:** Robert Clarke, Derrick A. Bennett, Sarah Parish, Petra Verhoef, Mariska Dötsch-Klerk, Mark Lathrop, Peng Xu, Børge G. Nordestgaard, Hilma Holm, Jemma C. Hopewell, Danish Saleheen, Toshihiro Tanaka, Sonia S. Anand, John C. Chambers, Marcus E. Kleber, Willem H. Ouwehand, Yoshiji Yamada, Clara Elbers, Bas Peters, Alexandre F. R. Stewart, Muredach M. Reilly, Barbara Thorand, Salim Yusuf, James C. Engert, Themistocles L. Assimes, Jaspal Kooner, John Danesh, Hugh Watkins, Nilesh J. Samani, Rory Collins, Richard Peto, for the MTHFR Studies Collaborative Group

**Affiliations:** 1 Clinical Trial Service Unit and Epidemiological Studies Unit (CTSU), University of Oxford, Oxford, United Kingdom; 2 Unilever Research and Development, Vlaardingen, The Netherlands; 3 Centre National de Genotypage, Evry, France; 4 Herlev Hospital Department of Clinical Biochemistry, University of Copenhagen, Copenhagen, Denmark; 5 deCODE Inc, Rejkavik, Iceland; 6 Department of Public Health and Primary Care, University of Cambridge, United Kingdom; 7 Center for Non-Communicable Diseases, Karachi, Pakistan; 8 RIKEN Centre for Genomic Medicine, Yokohama, Japan; 9 Population Health Research Institute, Hamilton, Canada; 10 Imperial College Faculty of Medicine, University of London, London, United Kingdom; 11 Luric Study Non-Profit LCC, University of Freiburg, Freiburg, Germany; 12 Department of Haematology, University of Cambridge, United Kingdom; 13 Mie University Life Science Research Center, Tsu, Japan; 14 Department of Medical Genetics, University of Utrecht, Utrecht, The Netherlands; 15 Utrecht Institute for Pharmaceutical Sciences, University of Utrecht, Utrecht, The Netherlands; 16 Heart Institute, University of Ottawa, Ottawa, Canada; 17 Cardiovascular Institute, University of Pennsylvania, Philadelphia, Pennsylvania, United States of America; 18 Helmholtz Zentrum, Institute of Epidemiology II, German Research Center for Environmental Health, Munich, Germany; 19 McGill University Health Centre, Montreal, Canada; 20 Department of Medicine, Stanford University School of Medicine, Stanford, California, United States of America; 21 Department of Cardiovascular Medicine, University of Oxford, United Kingdom; 22 Department of Cardiovascular Sciences, University of Leicester, United Kingdom; University of Bristol, United Kingdom

## Abstract

**Background:**

Moderately elevated blood levels of homocysteine are weakly correlated with coronary heart disease (CHD) risk, but causality remains uncertain. When folate levels are low, the TT genotype of the common C677T polymorphism (rs1801133) of the methylene tetrahydrofolate reductase gene (*MTHFR*) appreciably increases homocysteine levels, so “Mendelian randomization” studies using this variant as an instrumental variable could help test causality.

**Methods and Findings:**

Nineteen unpublished datasets were obtained (total 48,175 CHD cases and 67,961 controls) in which multiple genetic variants had been measured, including *MTHFR* C677T. These datasets did not include measurements of blood homocysteine, but homocysteine levels would be expected to be about 20% higher with TT than with CC genotype in the populations studied. In meta-analyses of these unpublished datasets, the case-control CHD odds ratio (OR) and 95% CI comparing TT versus CC homozygotes was 1.02 (0.98–1.07; *p* = 0.28) overall, and 1.01 (0.95–1.07) in unsupplemented low-folate populations. By contrast, in a slightly updated meta-analysis of the 86 published studies (28,617 CHD cases and 41,857 controls), the OR was 1.15 (1.09–1.21), significantly discrepant (*p* = 0.001) with the OR in the unpublished datasets. Within the meta-analysis of published studies, the OR was 1.12 (1.04–1.21) in the 14 larger studies (those with variance of log OR<0.05; total 13,119 cases) and 1.18 (1.09–1.28) in the 72 smaller ones (total 15,498 cases).

**Conclusions:**

The CI for the overall result from large unpublished datasets shows lifelong moderate homocysteine elevation has little or no effect on CHD. The discrepant overall result from previously published studies reflects publication bias or methodological problems.

*Please see later in the article for the Editors' Summary*

## Abstract 2

Robert Clarke and colleagues conduct a meta-analysis of unpublished datasets to examine the causal relationship between elevation of homocysteine levels in the blood and the risk of coronary heart disease. Their data suggest that an increase in homocysteine levels is not likely to result in an increase in risk of coronary heart disease.

## Editors' Summary

**Background:**

Coronary heart disease (CHD) is the leading cause of death among adults in developed countries. With age, fatty deposits (atherosclerotic plaques) coat the walls of the coronary arteries, the blood vessels that supply the heart with oxygen and nutrients. The resultant restriction of the heart's blood supply causes shortness of breath, angina (chest pains that are usually relieved by rest), and sometimes fatal heart attacks. Many established risk factors for CHD, including smoking, physical inactivity, being overweight, and eating a fat-rich diet, can be modified by lifestyle changes. Another possible modifiable risk factor for CHD is a high blood level of the amino acid homocysteine. Methylene tetrahydofolate reductase, which is encoded by the *MTHFR* gene, uses folate to break down and remove homocysteine so fortification of cereals with folate can reduce population homocysteine blood levels. Pooled results from prospective observational studies that have looked for an association between homocysteine levels and later development of CHD suggest that the reduction in homocysteine levels that can be achieved by folate supplementation is associated with an 11% lower CHD risk.

**Why Was This Study Done?:**

Prospective observational studies cannot prove that high homocysteine levels cause CHD because of confounding, the potential presence of other unknown shared characteristics that really cause CHD. However, an approach called “Mendelian randomization” can test whether high blood homocysteine causes CHD. A common genetic variant of the *MTHFR* gene—the C677T polymorphism—reduces MTHFR efficiency so TT homozygotes (individuals in whom both copies of the *MTHFR* gene have the nucleotide thymine at position 677; the human genome contains two copies of most genes) have 25% higher blood homocysteine levels than CC homozygotes. In meta-analyses (statistical pooling of the results of several studies) of published Mendelian randomized studies, TT homozygotes have a higher CHD risk than CC homozygotes. Because gene variants are inherited randomly, they are not subject to confounding, so this result suggests that high blood homocysteine causes CHD. But what if only Mendelian randomization studies that found an association have been published? Such publication bias would affect this aggregate result. Here, the researchers investigate the association of the *MTHFR* C677T polymorphism with CHD in unpublished datasets that have analyzed this polymorphism incidentally during other genetic studies.

**What Did the Researchers Do and Find?:**

The researchers obtained 19 unpublished datasets that contained data on the *MTHFR* C677T polymorphism in thousands of people with and without CHD. Meta-analysis of these datasets indicates that the excess CHD risk in TT homozygotes compared to CC homozygotes was 2% (much lower than predicted from the prospective observational studies), a nonsignificant difference (that is, it could have occurred by chance). When the probable folate status of the study populations (based on when national folic acid fortification legislation came into effect) was taken into account, there was still no evidence that TT homozygotes had an excess CHD risk. By contrast, in an updated meta-analysis of 86 published studies of the association of the polymorphism with CHD, the excess CHD risk in TT homozygotes compared to CC homozygotes was 15%. Finally, in a meta-analysis of randomized trials on the use of vitamin B supplements for homocysteine reduction, folate supplementation had no significant effect on the 5-year incidence of CHD.

**What Do These Findings Mean?:**

These analyses of unpublished datasets are consistent with lifelong moderate elevation of homocysteine levels having no significant effect on CHD risk. In other words, these findings indicate that circulating homocysteine levels within the normal range are not causally related to CHD risk. The meta-analysis of the randomized trials of folate supplementation also supports this conclusion. So why is there a discrepancy between these findings and those of meta-analyses of published Mendelian randomization studies? The discrepancy is too large to be dismissed as a chance finding, suggest the researchers, but could be the result of publication bias—some studies might have been prioritized for publication because of the positive nature of their results whereas the unpublished datasets used in this study would not have been affected by any failure to publish null results. Overall, these findings reveal a serious example of publication bias and argue against the use of folate supplements as a means of reducing CHD risk.

**Additional Information:**

Please access these Web sites via the online version of this summary at http://dx.doi.org/10.1371/journal.pmed.1001177.

- The American Heart Association provides information about CHD and tips on keeping the heart healthy; it also provides information on homocysteine, folic acid, and CHD, general information on supplements and heart health, and personal stories about CHD
- The UK National Health Service Choices website provides information about CHD, including personal stories about CHD
- Information is available from the British Heart Foundation on heart disease and keeping the heart healthy
- The US National Heart Lung and Blood Institute also provides information on CHD (in English and Spanish)
- MedlinePlus provides links to many other sources of information on CHD (in English and Spanish)
- Wikipedia has a page on Mendelian randomization (note: Wikipedia is a free online encyclopedia that anyone can edit; available in several languages)

## Introduction

Rare genetic defects that cause extremely high plasma homocysteine levels also cause coronary heart disease (CHD) [1]–[3]. It was therefore hypothesised that, even within the normal range of plasma homocysteine concentrations, higher levels might appreciably increase CHD risk [3]. Retrospective studies originally suggested a strong relationship, but subsequent prospective observational studies suggested weaker associations [3],[4]. A meta-analysis of prospective studies found that, after adjusting for known risk factors, 25% lower usual homocysteine level (achievable in many populations by fortification of cereals with folic acid) was associated with only about 11% (95% CI 4%–17%, *p*<0.001) lower CHD risk [4]. Although significant, the weak association represented by the lower confidence limit could be largely or wholly noncausal (as, for example, homocysteine might be associated with renal failure or other vascular risk factors, or might reflect preexisting atherosclerosis).

A meta-analysis of the randomized trials of folic acid, involving 37,485 individuals, reported that an average 25% reduction in homocysteine levels throughout a median follow-up of 5 y had no significant effect on major vascular events [5]. As the duration of treatment was only a few years, it has been suggested that more prolonged treatment might be protective against the onset of CHD [6]. Hence, reliable studies are needed of genetic variants that affect homocysteine levels throughout life.

The enzyme methylene tetrahydrofolate reductase, encoded by the *MTHFR* gene, uses folate to metabolise and thereby remove homocysteine [7]. The *MTHFR* C677T polymorphism (rs1801133) is common (T-allele frequency 15%–45% in many populations) and reduces enzyme efficiency. In many populations without folic acid fortification, individuals with TT genotype have about 20% higher homocysteine than those with the more common CC genotype [8],[9]. Individuals are, in effect, randomly allocated at conception to *MTHFR* genotype and, hence, to higher or lower lifelong homocysteine levels [9]. If the associations seen in prospective studies were largely causal, the 20% higher usual homocysteine in TT homozygotes would imply about 8% (95% CI 3%–13%) higher CHD risk.

Such Mendelian randomized studies of the associations of *MTHFR* genotype with CHD assess the effects of lifelong homocysteine differences and should not be materially affected by confounding if each study is of a reasonably homogeneous population (or if any population admixture can be adequately allowed for) [9]. Because the expected effect on risk is small, however, reliable assessment of it requires extremely large numbers of cases and strict avoidance of any potential sources of moderate bias, including publication bias. Previous meta-analyses just of the published studies [6],[10]–[12] have found, in aggregate, a highly significant but only moderately positive association of *MTHFR* genotype with CHD risk (odds ratio [OR] for TT versus CC genotype of 1.16 in the most recent report [6]), but opinions differ as to whether publication bias could explain away this aggregate result [6],[10]–[12]. As genotyping has become less expensive, large datasets have started to emerge from genome-wide association (GWA) and gene-chip studies in which *MTHFR* C677T was analyzed largely incidentally as one of many thousands, or even hundreds of thousands, of polymorphisms included on standard genotyping platforms. In none of these studies had the *MTHFR* OR been published. The *MTHFR* variant, together with multiple other polymorphisms, had also been genotyped in some large previously unpublished case-control studies.

We report meta-analyses of the association of the *MTHFR* C677T polymorphism with CHD risk in these unpublished datasets and contrast them with meta-analyses of the published studies of this polymorphism and an updated meta-analysis of the CHD results in the randomized trials of B-vitamins for homocysteine reduction. In some populations, introduction of folic acid fortification around the mid-1990s changed mean plasma folate levels appreciably [13]. Previous meta-analyses did not take account of secular changes in folate [6],[14] when considering associations of *MTHFR* genotype with CHD [6],[10]–[12]. We subdivide our findings by the approximate effects of the C677T polymorphism on homocysteine levels expected in the different populations studied.

## Methods

### Unpublished Studies of *MTHFR* and CHD

Previously unpublished CHD case-control results were sought from large-scale genotyping datasets: from the two large collaborative consortia, C4D [15] and CARDIOGRAM [16], convened to conduct meta-analyses with maximum power to detect novel susceptibility variants for CHD (all members with data on rs1801133 collaborated); and from other affiliated studies, including the ISIS case-control study [17], the INTERHEART study [18], and from the investigators of large-scale genotyping studies in Japan (all of whom collaborated). The genotyping panels ranged in panel size from 67 polymorphisms to hundreds of thousands of polymorphisms, and results were adjusted internally where investigators were able to do so for population admixture and familial clustering. In none of these studies was the *MTHFR* polymorphism of primary interest, and their *MTHFR* CHD ORs had, when we requested data, not yet been reported, so these are referred to as unpublished datasets. Some of these studies had, in publications on their positive findings, implied that any association with *MTHFR* was nonsignificant (at some significance level). CHD was defined as death from CHD, myocardial infarction (by WHO MONICA criteria [19]), or angiographic stenosis (involving at least 50% of a major coronary artery). Almost all CHD cases (when bled) had nonfatal myocardial infarction. No unpublished studies (and few published ones) were of angiographic CHD only. We identified a total of 48,175 CHD cases and 67,961 controls in these unpublished datasets (Table S1 in Text S1).

### Published Studies of *MTHFR* and CHD

Previous meta-analyses of published studies [6],[9],[10]–[12],[14],[20] were updated by searching the electronic literature (PubMed, Current Contents, and HuGENet) seeking relevant studies published before 2010 using search terms “*MTHFR*” and “coronary heart disease” or “coronary stenosis” or “myocardial infarction,” or by hand-searching reference lists of identified studies, review articles, and previous meta-analyses, and by contacting investigators. Studies were included if they had been published as articles or letters in peer-reviewed journals, had a case-control design or a nested case-control design within a prospective study, and reported their results by genotype. If two reports of the same study were found, only the one based on the larger dataset was used. For published case-control studies, individual participant datasets were sought; where unavailable, tabular data sufficed (checked if possible with investigators). We identified a total of 28,617 cases and 41,857 controls in 86 published case-control studies (Figure S1; Table S2 in Text S1).

### *MTHFR* and Homocysteine Levels

Of the 86 published case-control studies, only 37 yielded data on normal homocysteine levels (in a total of 14,774 controls). None of the unpublished datasets yielded such data, as the investigations were not particularly concerned with homocysteine. Additional eligible studies were identified by searching the electronic literature (PubMed, Current Contents, and HuGENet) using the search terms “*MTHFR*” and “homocysteine” or “total homocysteine” for relevant reports published before 2010, by hand searching reference lists of original studies and review articles (including meta-analyses) on this topic seeking data on the *MTHFR* genotypes and plasma homocysteine levels in disease-free individuals. The search criteria identified 53,595 other disease-free individuals with homocysteine data in 33 other *MTHFR* publications, yielding a total of 70 published studies of the biochemical effects of *MTHFR* genotype on homocysteine (Figure S1; Table S3 in Text S1).

### Categorization of *MTHFR* Studies by Probable Folate Status

Folate status was generally unknown in the *MTHFR* studies. As a surrogate for it, these *MTHFR* studies were classified by study place and time into five probable folate status categories, on the basis of when national legislation permitting or requiring folic acid fortification came into effect (Appendix S1 in Text S1). Population surveys of folate status published before the end of 2009 of healthy individuals, including controls from case-control studies or participants in randomized trials in healthy volunteers were identified from previous meta-analyses [4],[11],[21] and by searching the electronic literature using the search terms “homocysteine,” “total homocysteine,” “folic acid,” “folate,” “B-vitamins,” “folic acid fortification,” and “population or nutrition surveys.” The search criteria identified 81 population-based surveys that reported mean serum folate levels in general population samples of >100 people (Figure S2; Table S4 in Text S1). Mean folate levels were estimated for each of these five categories on the basis of secular and geographic trends in folic acid fortification policies (Appendix S1 in Text S1).

### Randomized Folate Trials

Finally, we updated a previous meta-analysis [5] of seven large-scale placebo-controlled trials assessing the effects on cardiovascular disease of lowering homocysteine with B-vitamins by adding three trials [22]–[24] that reported their results after publication of the meta-analysis (Table S5 in Text S1). The additional trials were identified by searching the electronic literature using search terms “cardiovascular disease,” “coronary heart disease,” “coronary stenosis,” “myocardial infarction” and “randomized controlled trial,” “clinical trial,” and “folic acid” or “B-vitamins.” As in the original meta-analysis [5], additional randomized trials were eligible if (i) they involved a double-blind randomized comparison of B-vitamin supplements containing folic acid versus placebo for the prevention of vascular disease; (ii) the relevant treatment arms differed only with respect to the homocysteine-lowering intervention; and (iii) the trial involved ≥1,000 participants with treatment duration of ≥1 y.

### Statistical Methods

Mean folate levels and mean log homocysteine by genotype were estimated from individual participant data where available, or from published reports. In calculating these means we sought to give all individuals similar weight, so large studies contribute proportionally more than small ones. (Random effects models were not used, as they can give undue weight to individuals in smaller studies [25].) The homocysteine difference between TT and CC genotypes was estimated from linear regression (stratified by study) of log homocysteine on genotype in heterozygotes [26],[27]. The CHD OR for TT versus CC genotype (OR) was estimated by logistic regression, stratified by study; this yields an approximately inverse-variance-weighted average of the log OR in each study. In the PROCARDIS study, which included both related and unrelated cases and controls, allowance for familial clustering was made, which slightly increased the variance estimate [15]. In the LOLIPOP and PROMIS studies of South Asians also, the CHD OR for TT versus CC genotypes was estimated after correction for population admixture (to avoid false positive association due to population stratification) using adjustment for principal components involving the results of random genetic markers within that study [15], which was not possible in the published studies. Details of the methods used to estimate nonpublication bias are shown in Appendix S2 in Text S1. Heterogeneity was assessed using chi-squared tests [28], also citing *I* ^2^ = 100%(1−[degrees of freedom]/[chi-squared test statistic]) [29]. CIs are 95%, except where specified as 99% to allow for multiple comparisons. Analyses used SAS version 9.1.

## Results

**Figure 1:**
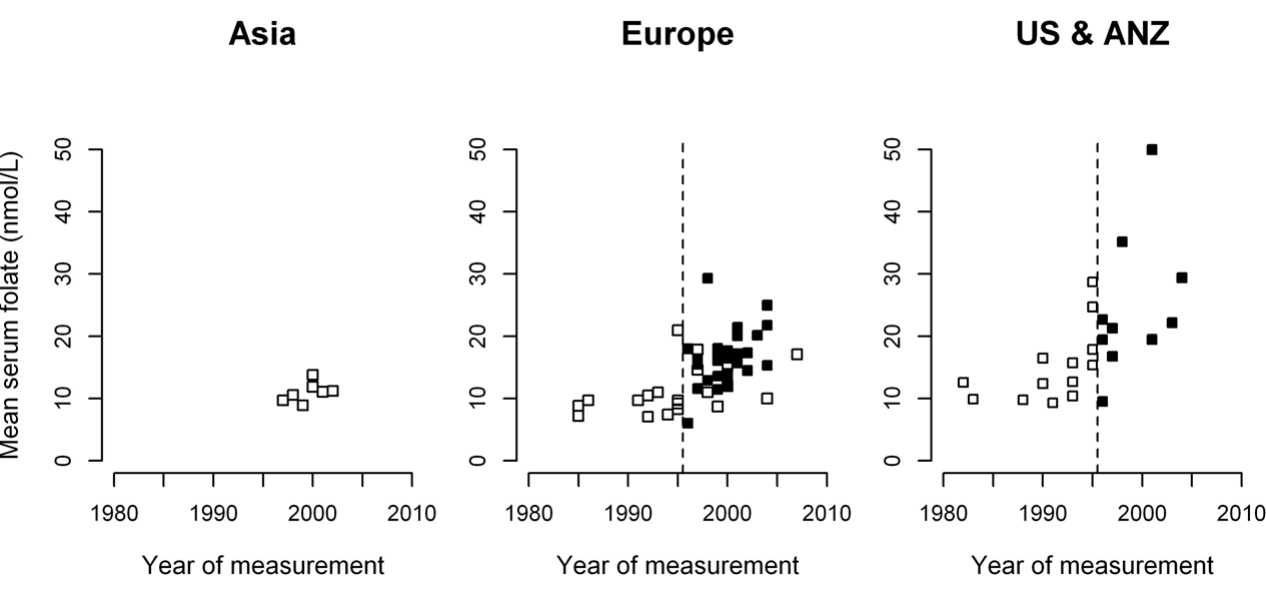
Mean serum folate concentrations in 81 population surveys, by calendar year and region. White squares, no folate supplementation; black squares, after folate supplementation; broken vertical line, 1995–1996, when folate supplementation began in the United States, Canada, Australia, New Zealand (US & ANZ), and some but not all European countries. No Asian surveys were in supplemented populations.

Figure 1 plots mean folate levels by calendar year in 81 population surveys (total 200,103 participants), categorising the surveys by study place (Asia, Europe or North America and Australasia [US & ANZ]) and time (before or after national folate supplementation began). Asian surveys were all in unsupplemented populations, so Figure 1 defines only five probable folate status categories. Table 1 gives the mean folate levels in each category. Although assay methods may have varied, there appeared to be similarly low folate levels in the Asian and unsupplemented European populations (11.0 and 11.9 nmol/l), intermediate folate levels in the supplemented European and unsupplemented US and ANZ populations (18.2 and 20.8 nmol/l), and high folate levels in supplemented US and ANZ populations (33.3 nmol/l). Thus, there are only two low-folate unsupplemented categories.

**Table 1:** Relevance in population surveys of study place and time to (i) the mean general population serum folate level, and (ii) the excess plasma homocysteine level in the TT versus CC *MTHFR* C677T genotype.

Region, and Whether after Folate Supplementation	Surveys of Folate Levels			Studies of *MTHFR* C677T Genotype and Plasma Homocysteine		
	Folate Surveys	*n* people	Mean (SE) Serum Folate Concentration, nmol/l^a^	Homocysteine *MTHFR* Studies	*n* People	Percent Higher Homocysteine, TT Versus CC (and 99% CI)^b^
Asia^c^ no supplementation	7	4,841	11.0 (0.014)	15	6,553	25 (21–30)
Europe, presupplementation	21	31,767	11.9 (0.006)	14	24,199	21 (19–24)
Europe, post-supplementation	30	13,504	18.2 (0.009)	25	8,702	18 (15–22)
US & ANZ, presupplementation	13	57,104	20.8 (0.004)	8	26,853	13 (11–15)
US & ANZ, post-supplementation	10	92,887	33.3 (0.003)	8	2,062	7 (2–13)
All regions and time periods	81	200,103	24.8 (0.002)	70	68,369	18 (17–19)

a Mean folate levels average all who were surveyed; SE denotes the standard error due only to within-survey variation. Between-survey variation in folate levels is illustrated in Figure 1.

b From inverse-variance-weighted averages of within-study differences in log homocysteine; Figure S1, Table S2 in Text S1.

c Mainly of Japanese, Chinese, or Korean populations; none of South Asians.

Homocysteine differences by *MTHFR* genotype are also given in Table 1, based on 70 biochemical studies of *MTHFR* genotype and homocysteine in the general population (total 68,369 participants, mostly Caucasian or East Asian). These analyses of within-study percentage differences in homocysteine levels between TT and CC genotypes (Figure S3) should be little affected by any variation in homocysteine assay methods. The TT versus CC homocysteine difference appears to have been only moderately affected by folate supplementation, but was appreciably greater in Asia and Europe than in the US & ANZ (although the TT versus CC homocysteine difference in US & ANZ after folate supplementation had a wide CI and is not reliably known). Differences in homocysteine between the CT and CC genotypes were only about a quarter as great as those between the homozygous TT and CC genotypes (Figure S3). Tables S3 and S4 in Text S1 give separately each survey of folate levels and each study of *MTHFR* genotype and homocysteine, and Tables S1, S2 in Text S1 give separately each case-control study result. Among the controls there was substantial variation in genotype frequencies (ratio of TT to CC 0.03–0.04 in South Asians, 0.2–0.3 in northern Europe, 0.4 or more in Japan, and 0.7 in Italy), illustrating the potential for bias from population substructure.

**Figure 2:**
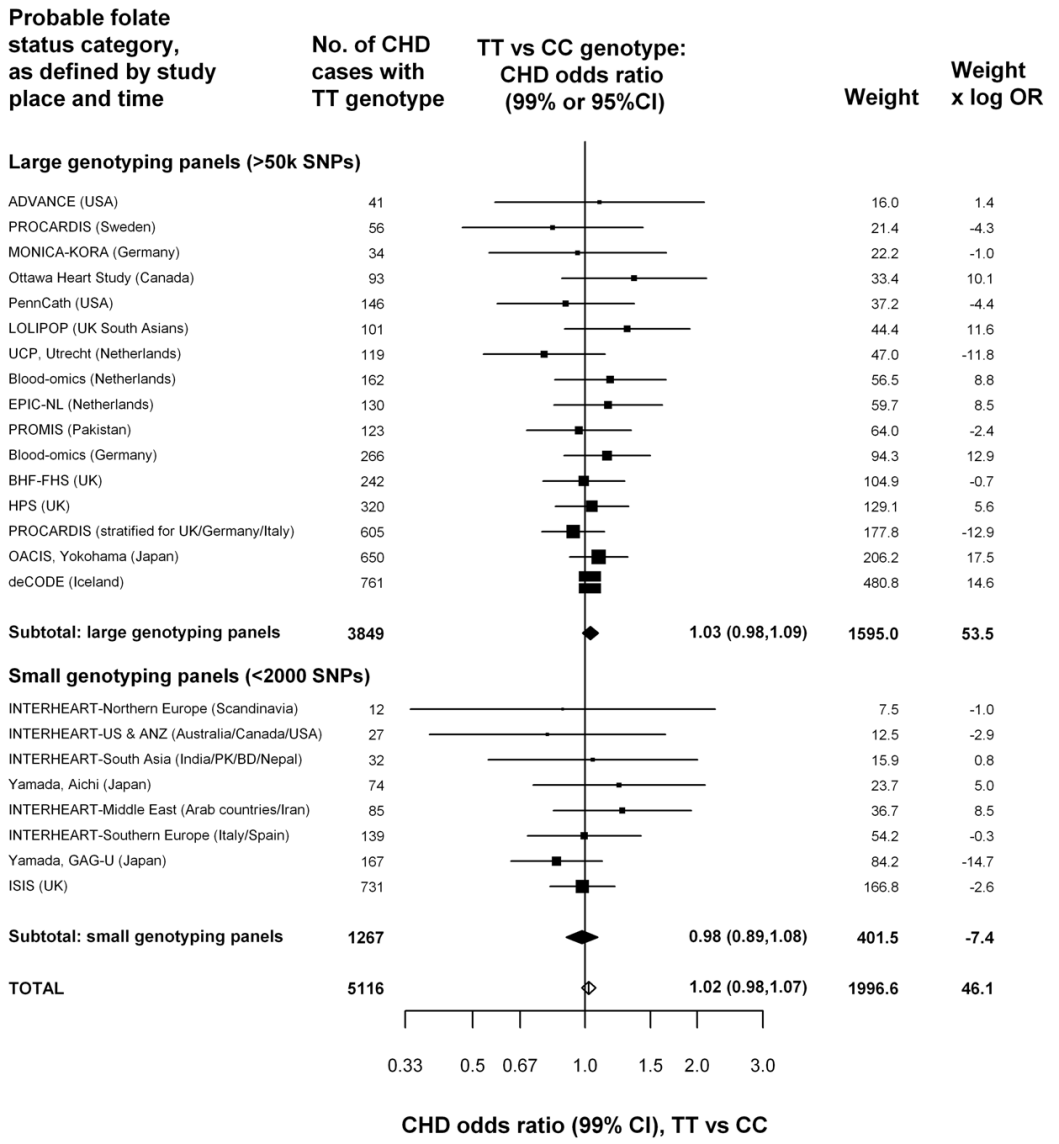
Homozygote CHD OR (TT versus CC *MTHFR* C677T genotype) in 19 unpublished datasets, yielding 24 parts that are classified by genotyping panel size. For these datasets, being unpublished introduces a negligible bias (less than 0.3% for each OR and about 0.1% for the overall OR: eAppendix 1). Black squares indicate OR (with areas inversely proportional to the variance of log OR), and horizontal lines indicate 99% CIs. The subtotals and their 99% CIs are indicated by black diamonds. The overall OR and its 95% CI is indicated by a white diamond. The weight (defined as the inverse of the variance of the maximum likelihood estimate of the log OR) and the product of the weight times OR indicates how much each study has contributed to the subtotals and totals. Because the weights and products are approximately additive, they can be used to estimate the effects of ignoring particular studies, or of grouping studies in different ways.

Our case-control analyses of *MTHFR* genotype and CHD risk compare TT versus CC homozygotes, as this is the comparison that involves the greatest homocysteine contrast. The findings for CHD risk in the unpublished datasets are given in Figure 2, subdivided by the number of variants examined (i.e., genotyping panel size). Overall, there would have been about a 20% excess homocysteine associated with the TT versus the CC genotype, but the excess CHD risk associated with the TT versus the CC genotype was only 2%, was not significant (OR = 1.02, 95% CI 0.98–1.07, *p* = 0.28), and was similar in the datasets with larger and small genotyping panel sizes. Any null bias from nonpublication would have biased the expected log OR in the aggregate of all unpublished studies downwards by only about 0.001 (0.003 in the small-panel studies and 0.0002 in the large-panel studies: Appendix S2 in Text S1), thereby multiplying the overall OR by 0.999, which is negligible.

**Figure 3:**
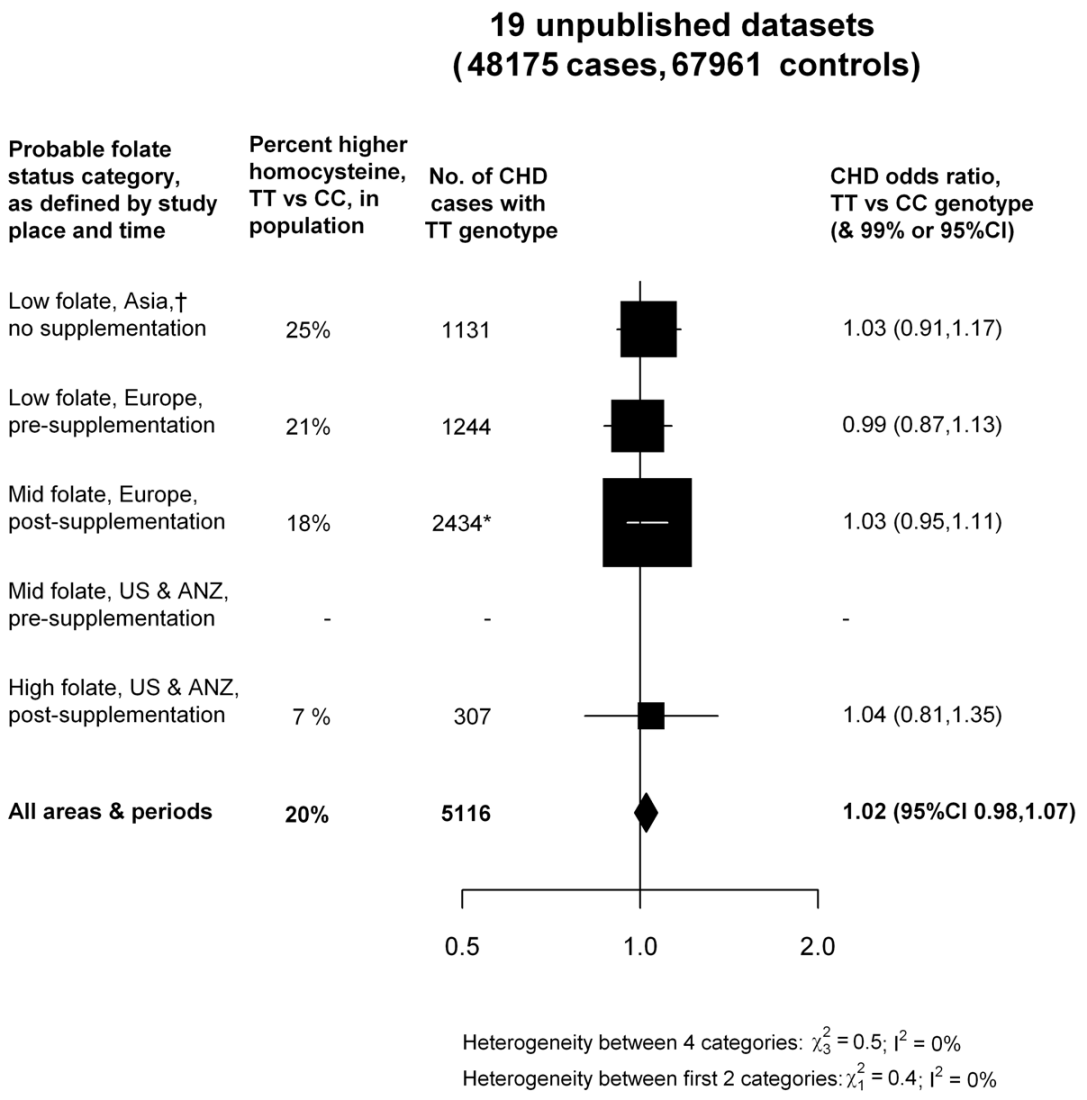
Homozygote CHD OR (TT versus CC *MTHFR* C677T genotype) in each probable folate status category, from meta-analyses of 19 unpublished datasets (all large). Average homocysteine difference (in the non-CHD general population) for all areas and periods is weighted in proportion to the numbers of TT CHD cases in all 19 unpublished datasets. Nonpublication involves negligible bias: Appendix S2 in Text S1.

Figure 3 categorizes these results by the probable folate status of the populations studied. Half the evidence was from low-folate unsupplemented populations in Asia or Europe. But, even if attention is restricted to these populations (where the excess of homocysteine associated with the TT versus CC genotype would have been somewhat greater than elsewhere), there was still no evidence that the TT genotype was associated with any excess risk of CHD (OR = 1.01: 1.03 in low-folate Asia, 0.99 in low-folate Europe; Figure 3). As the homocysteine difference between CT and CC genotypes is only about a quarter of that between TT and CC genotypes, inclusion of the CT results does not materially alter these findings (Figure S4). Thus, the aggregated results from the 19 unpublished datasets suggested little or no hazard, even in unsupplemented low-folate populations.

**Figure 4:**
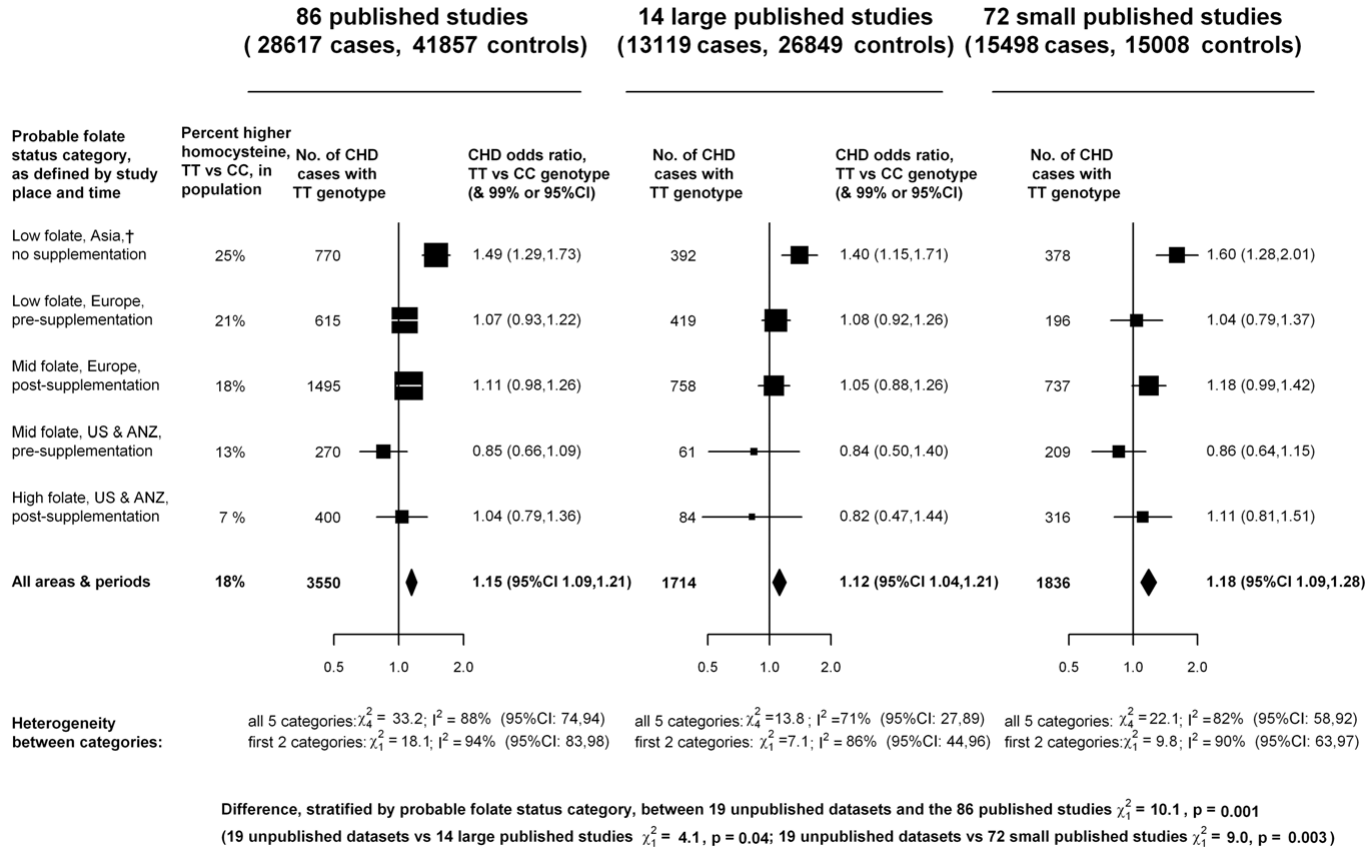
Homozygote CHD OR (TT versus CC *MTHFR* C677T genotype) in each probable folate status category, from meta-analyses of 86 published studies, 14 large (i.e., variance of log OR less than 0.05) and 72 smaller studies. Black squares indicate OR (with areas inversely proportional to the variance of log OR in each subdivision), and horizontal lines indicate 99% CIs. The overall OR and its 95% CI are indicated by a black diamond. Average homocysteine difference (in the non-CHD general population) for all areas and periods is weighted in proportion to the numbers of TT CHD cases in all 86 studies.

In contrast, the aggregated TT versus CC results from the 86 published studies (total 28,617 cases and 41,857 controls: Figure 4; Figure S5) suggested a 15% excess risk of CHD (OR 1.15, 95% CI 1.09–1.21), which is significantly discrepant (*p* = 0.001) with the results from the unpublished datasets (Figure 2). Larger studies may be less prone than smaller ones to selective publication based on their findings and may also be less prone to other, less clearly recognizable, methodological problems (and, publication bias may involve not only random but also any systematic errors due to preferential publication of positive results) [30]. In Figure S5, the CHD ORs in each of the 86 published studies are therefore ordered by study size (as defined by the variance of the log OR). Figure 4 indicates that, although the 72 smaller published studies contributed most to the suggestion of increased risk (OR = 1.18), the 14 larger published studies, which typically had >250 cases and >250 controls, also contributed to some extent (OR = 1.12).

Of these large studies, only two, both from Japan, suggested significantly increased risk. None of the others did, including those from low-folate Europe, where TT versus CC homocysteine differences were probably similar to those in Japan (Figure S3). The large published and unpublished Japanese studies are described separately in Table S6 in Text S1; in these, there appeared to be substantial heterogeneity in the TT and CC genotype frequencies (the odds, TT/CC, varied from 0.23 to 0.68 in controls), which makes it difficult to interpret the findings. (All these studies were located in mainland Japan, where there is little ethnic heterogeneity, so no large differences in genotype frequency would be expected [31].) The large published studies in all other populations, like the unpublished datasets, indicated no material effect between homozygote genotype and CHD risk.

Overall, almost half of the cases in the published CHD studies also had data on homocysteine, but those in the only two large studies with significantly increased risk did not. Hence, when analyses were restricted to the subset with homocysteine no significant association between TT versus CC genotype and CHD risk remained (unpublished data).

**Figure 5:**
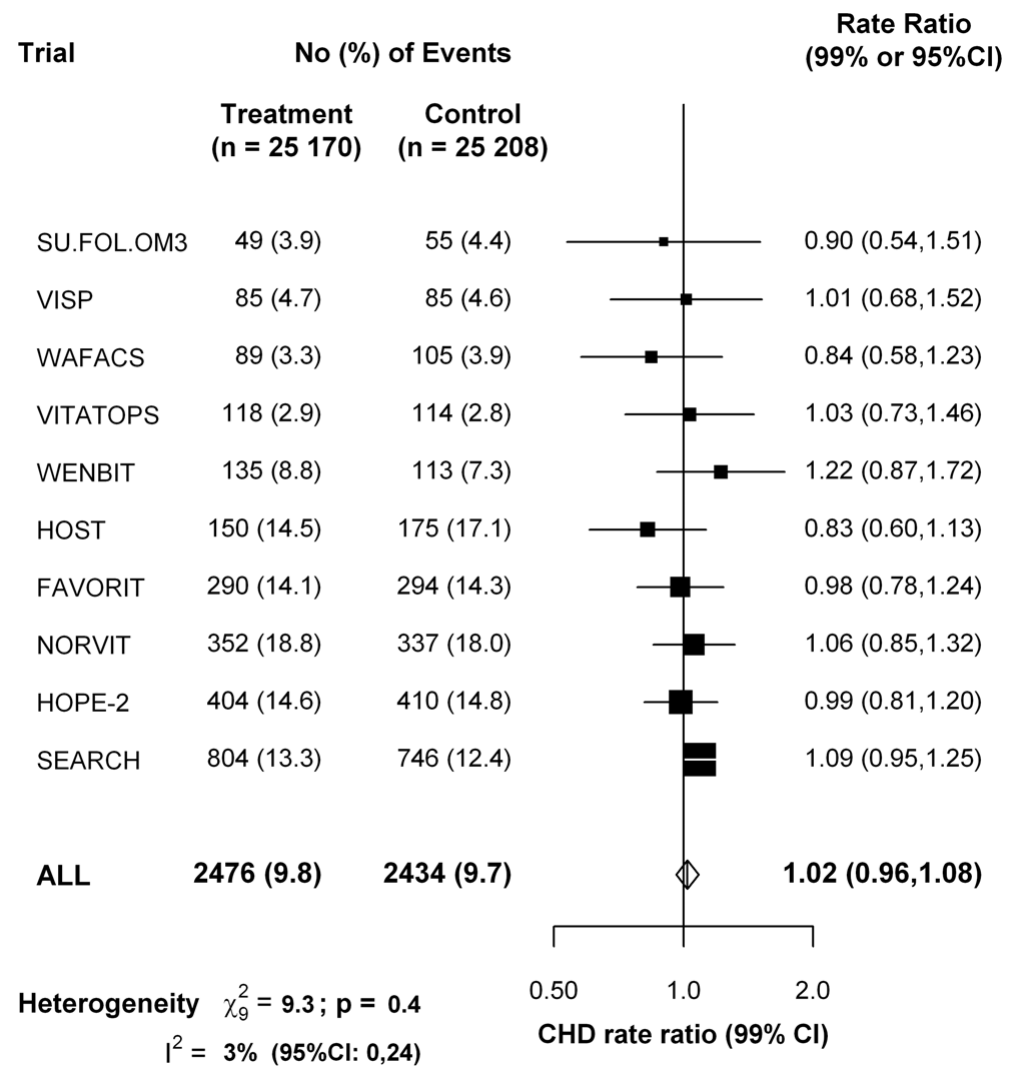
Effects of folic acid on major coronary events (nonfatal myocardial infarction or coronary death) in a meta-analysis of the published results of all large randomized trials of homocysteine reduction. Data for the VITATOPS trial are for myocardial infarction only. Data for FAVORIT are for all cardiovascular disease outcomes. Symbols and conventions as in Figure 2.

For the ten large trials of B-vitamins for homocysteine reduction (Table S5 in Text S1), Figure 5 shows that folate supplementation (which reduces normal homocysteine levels by about 25%) had little or no effect on the 5-y incidence of CHD incidence (rate ratio, folate versus placebo, 1.02, 95% CI 0.96–1.08).

## Discussion

The present meta-analyses of unpublished datasets involving 48,175 cases and 67,961 controls finds no evidence of an increased risk of CHD in TT versus CC homozygotes for the *MTHFR* C677T polymorphism, either in all such datasets or in those from unsupplemented low-folate populations. This null result is not materially affected by publication bias and is significantly discrepant with the moderately positive association found in our meta-analysis of 86 published studies of this question, or, equivalently, in other recent meta-analyses of published studies [6],[10]–[12].

Although publication bias (involving not only random errors but also any systematic errors in particular studies) may well have appreciably affected the meta-analyses of published studies, nonpublication bias (i.e., failure to publish null results) should have had a negligible effect on the present meta-analyses of unpublished studies. For ORs of the magnitude that may be plausible for *MTHFR* (i.e., about 1.08), the probability of a result from a large SNP panel study reaching statistical significance after allowance for multiple testing can be shown to be negligible (i.e., biasing the overall log OR by only about 0.001; Appendix S2 in Text S1). In these datasets, the TT versus CC comparison involves a nonsignificant excess CHD risk of only about 2% in all populations and 1% in low-folate unsupplemented populations (both with upper confidence limit 7%). Consistent with the null results of the folate trials, the results of the present meta-analyses of unpublished *MTHFR* studies provide no evidence for an association of life-long moderate elevations in homocysteine levels with CHD risk and support the suggestion [12] that the associations observed in meta-analyses of previously published *MTHFR* studies may be an artefact of publication bias.

The discrepancy between the overall results in the unpublished and the published datasets is too extreme to be plausibly dismissed as a chance finding (as is the discrepancy between the published results in low-folate Europe and Japan, which refutes the suggestion that differences in folate supplementation could explain the differences between Japanese and other published studies). Some studies, particularly if small, might have been prioritised for publication by investigators, referees, or editors according to the positivity of their results [30], and some may have been liable to other methodological problems that bias the average of all results. To avoid such biases, we chiefly emphasise the new results from the previously unpublished datasets. These show little or no hazard in Japan or elsewhere from moderate lifelong elevation of normal homocysteine levels.

The magnitude of the effect of publication bias is substantial and in addition to distorting the association of *MTHFR* with CHD in published studies, publication bias may also help explain the discrepant findings recently reported for *MTHFR* and stroke [32].

Genetic epidemiology of the effects of common polymorphisms on common diseases is increasingly dominated by consortia of GWA studies with tens of thousands of cases and large panels of tens or hundreds of thousands of polymorphisms [15],[16]. Thus, GWA (or other large panel genotyping) studies offer the possibility of avoiding unduly data-dependent emphasis on particular studies or on particular genetic loci and of making sophisticated allowance for population admixture. (Such allowance was not possible in the published studies and was available to us from only some of the unpublished datasets [15].) Although there is little evidence of significant population admixture in mainland Japan [31], the control frequency of the T allele varied somewhat across the Japanese case-control studies (0.33–0.45, Table S6 in Text S1), perhaps because variation in genotyping methods can affect *MTHFR* C677T genotype calls. As these small differences in T-allele frequency correspond to substantial differences in the TT/CC odds (Table S5 in Text S1), they reinforce the potential importance of cases and controls being blindly genotyped (assayed, called, and quality-control filtered) together, particularly for a polymorphism such as *MTHFR* C677T that varies in frequency between populations and does not have a substantial effect on risk.

The Mendelian randomization approach to assessing the effects of a particular biochemical factor such as homocysteine assumes no relevant pleiotropic effects of the genetic variant on other factors [33],[34] (whereas, for example, the TT genotype does also slightly affect folate levels) [35]. Large randomized trials of folate supplementation also provide an independent test of the causal relevance of homocysteine (assuming no material effects of folate on CHD except via homocysteine). A meta-analysis of 10 trials involving 50,378 participants had little or no effect on the 5-y incidence of CHD (rate ratio, folate versus placebo, 1.02, 95% CI 0.96–1.08). The null result from the folic acid trials is now directly reinforced by this Mendelian randomization meta-analysis of unpublished genetic epidemiology datasets, which is not materially affected by publication bias, involves large numbers of relevant outcomes, and shows no evidence that even a lifelong 20% difference in plasma homocysteine (within the normal range) meaningfully effects CHD risk.

## Supporting Information

Figure S1 **Screening and selection of articles for** ***MTHFR*** **and CHD risk and** ***MTHFR*** **and homocysteine levels.**
(TIF)

Figure S2 **Screening and selection of population surveys of folate status.**
(TIF)

Figure S3 **Percent higher homocysteine by** ***MTHFR*** **C677T genotype in 70 biochemical studies of non-CHD populations.** Subtotal results are from inverse-variance-weighted averages of within-study differences in log homocysteine, so the 95% CIs for them (solid diamonds) reflect only the within-study variation; other CIs are 99% CIs.
(TIF)

Figure S4 **CHD OR (OR, TT versus CC** ***MTHFR*** **C677T genotype) from CC/CT/TT results in 19 unpublished datasets, yielding 24 parts that are classified by probable folate status category: maximum likelihood estimate, assuming that the underlying log OR for CT/CC is 0.25 times that for TT/CC.** Black squares indicate OR, and horizontal lines indicate 99% CIs. The subtotals and their 99% CIs are indicated by black diamonds. The overall OR and its 95% CI is indicated by a white diamond. The weight (defined as the inverse of the variance of the maximum likelihood estimate of the log OR) and the product of the weight times OR indicates how much each study has contributed to the subtotals and totals.
(TIF)

Figure S5 **CHD OR for** ***MTHFR*** **TT versus CC genotype in 86 published studies, from Table S4, classified by probable folate status category and sorted by effective study size (i.e., variance of log OR, for which the cutoff 0.05 is indicated by dashed lines).** Weight is the inverse of the variance of the maximum likelihood estimate of the log OR. Additivity of the weights is therefore only approximate. NB, presupplementation Europe subtotal allows for the common control group in Frederiksen-Prospective (P) and Frederiksen-Case-Control (CC). 95% CIs for total; other CIs are 99%.
(TIF)

Text S1 **Webmaterial for homocysteine and coronary heart disease: meta-analysis of** ***MTHFR*** **case-control studies, avoiding publication bias.**
(PDF)

## Abbreviations

CHD: coronary heart disease
GWA: genome-wide association
*MTHFR*: methylene tetrahydrofolate reductase
OR: odds ratio
