# Supplementary figures and images for: Homocysteine and Coronary Heart Disease: Meta-analysis of MTHFR Case-Control Studies, Avoiding Publication Bias
Source: PLoS Med. 2012 Feb 21;9(2):e1001177. doi: 10.1371/journal.pmed.1001177 (PMC3283559; doi:10.1371/journal.pmed.1001177)

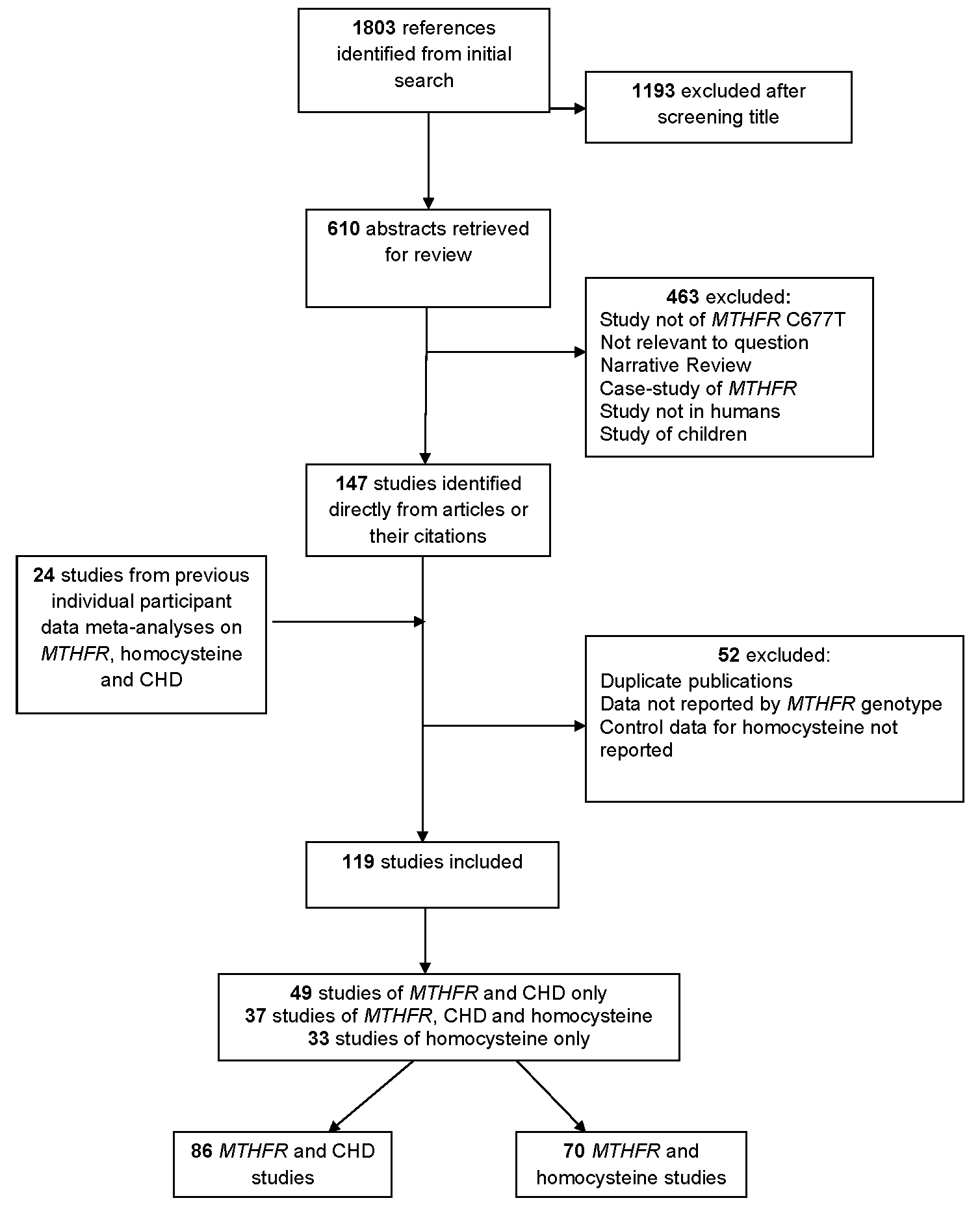

Supplement: Figure S1 — Screening and selection of articles for MTHFR and CHD risk and MTHFR and homocysteine levels. (TIF) [file pmed.1001177.s001.tif]

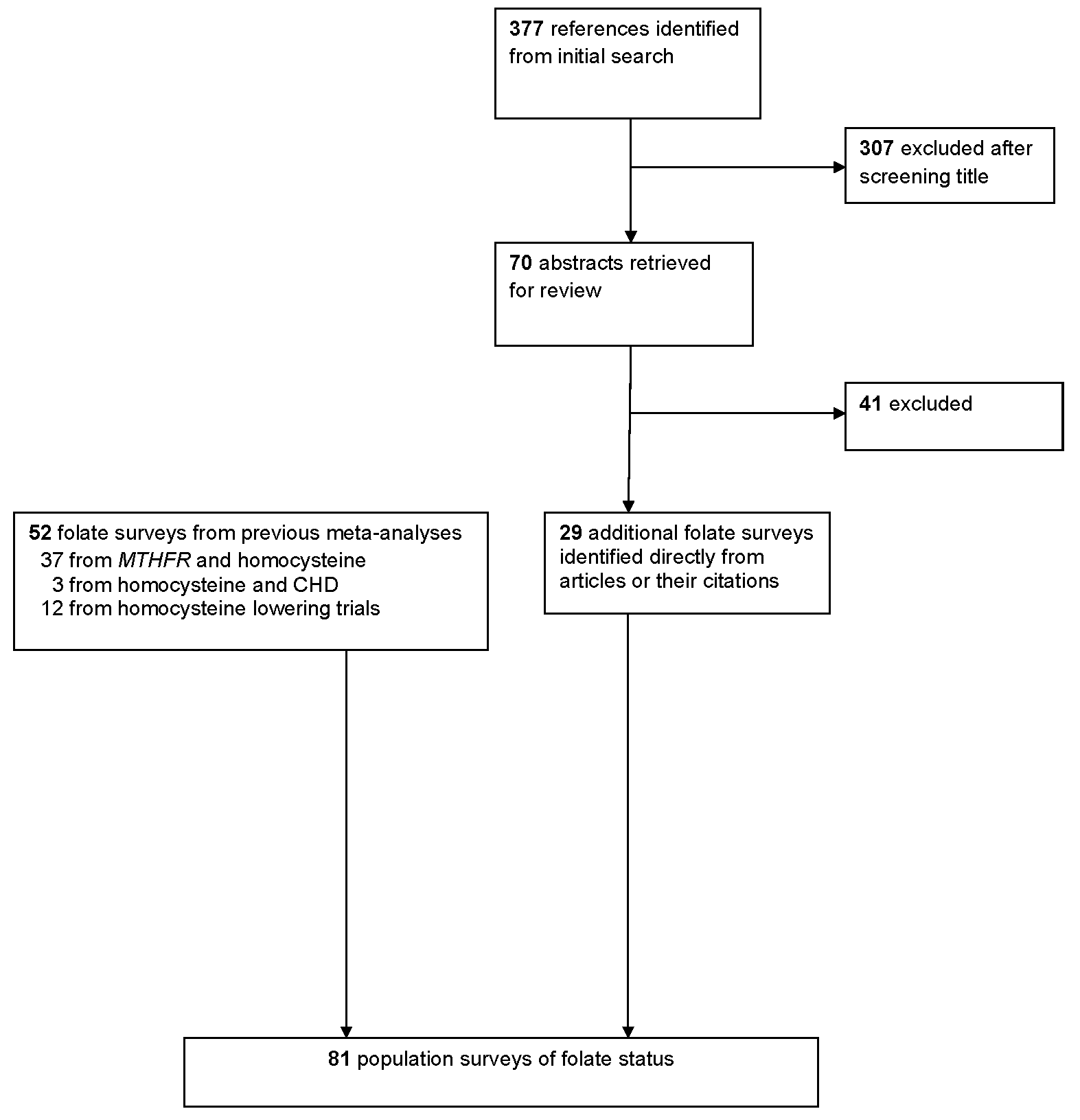

Supplement: Figure S2 — Screening and selection of population surveys of folate status. (TIF) [file pmed.1001177.s002.tif]

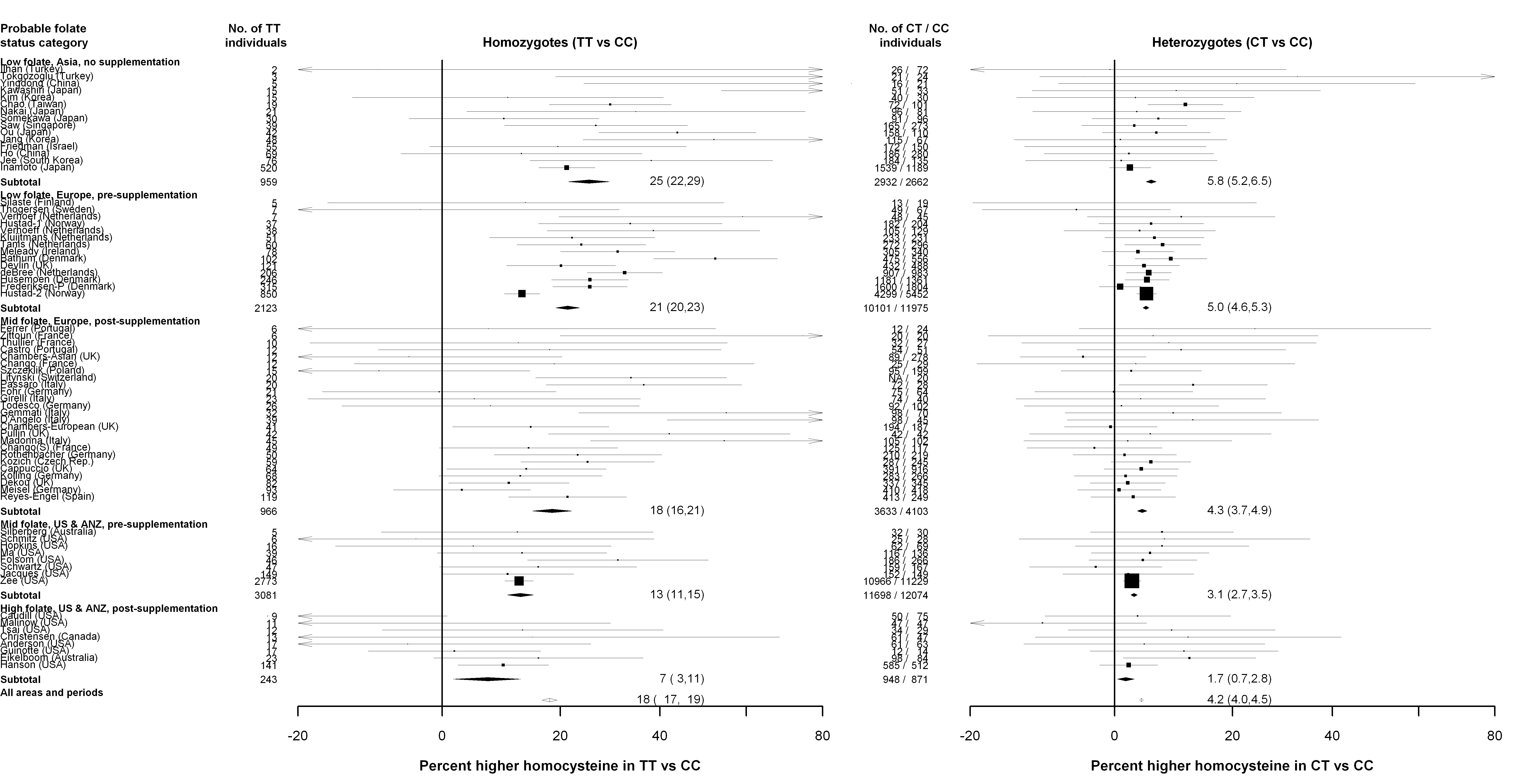

Supplement: Figure S3 — Percent higher homocysteine by MTHFR C677T genotype in 70 biochemical studies of non-CHD populations. Subtotal results are from inverse-variance-weighted averages of within-study differences in log homocysteine, so the 95% CIs for them (solid diamonds) reflect only the within-study variation; other CIs are 99% CIs. (TIF) [file pmed.1001177.s003.tif]

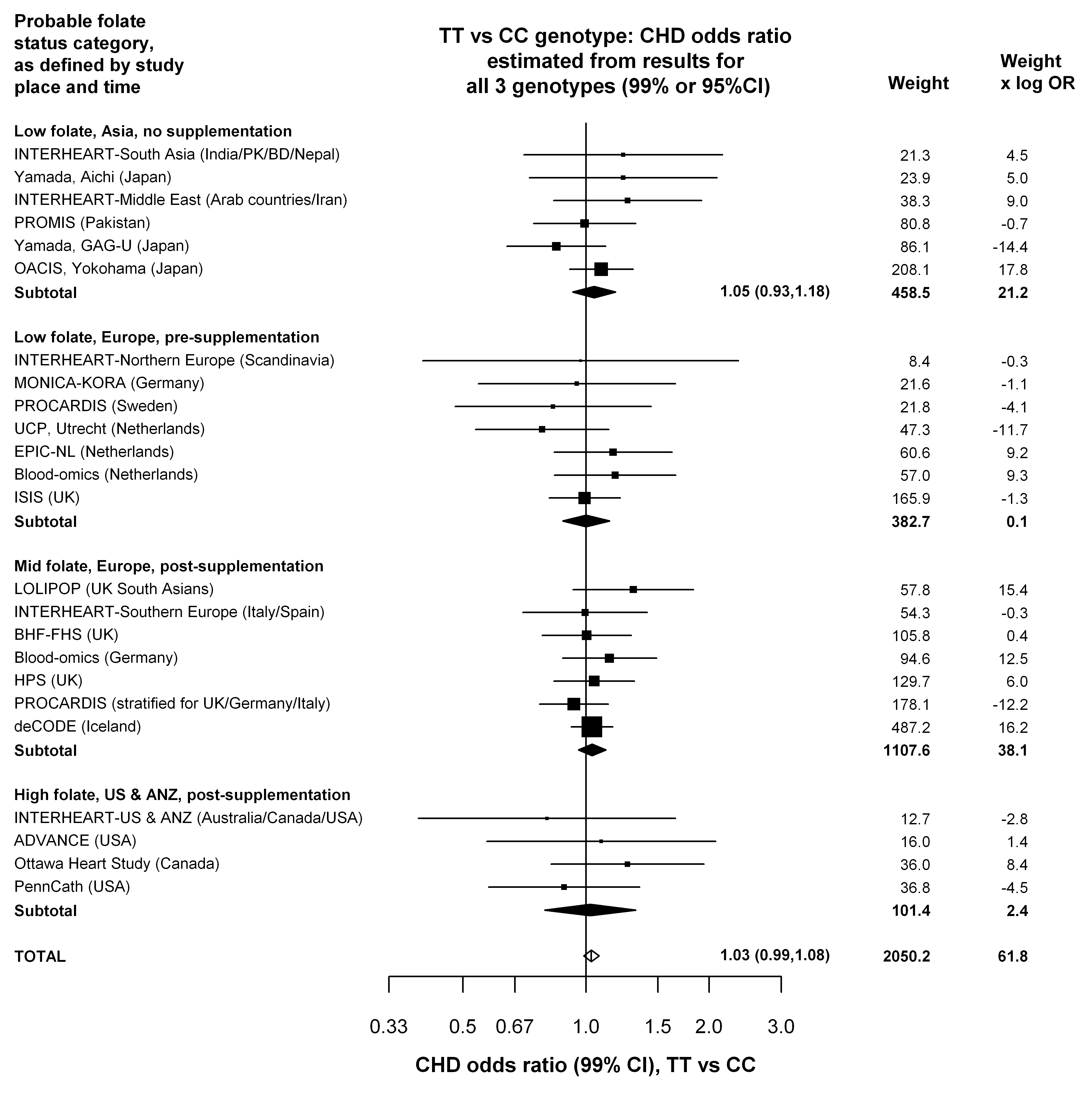

Supplement: Figure S4 — CHD OR (OR, TT versus CC MTHFR C677T genotype) from CC/CT/TT results in 19 unpublished datasets, yielding 24 parts that are classified by probable folate status category: maximum likelihood estimate, assuming that the underlying log OR for CT/CC is 0.25 times that for TT/CC. Black squares indicate OR, and horizontal lines indicate 99% CIs. The subtotals and their 99% CIs are indicated by black diamonds. The overall OR and its 95% CI is indicated by a white diamond. The weight (defined as the inverse of the variance of the maximum likelihood estimate of the log OR) and the product of the weight times OR indicates how much each study has contributed to the subtotals and totals. (TIF) [file pmed.1001177.s004.tif]

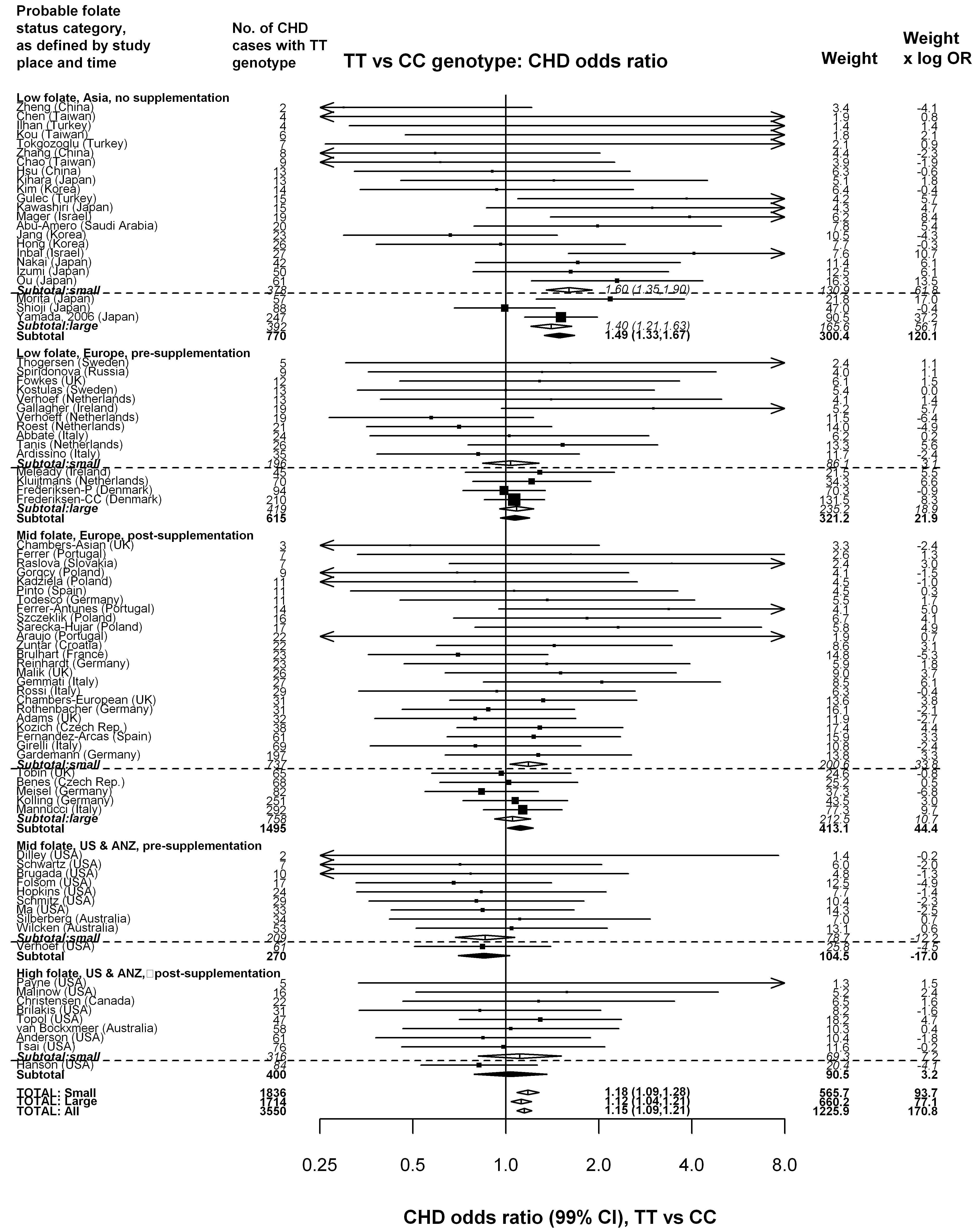

Supplement: Figure S5 — CHD OR for MTHFR TT versus CC genotype in 86 published studies, from Table S4, classified by probable folate status category and sorted by effective study size (i.e., variance of log OR, for which the cutoff 0.05 is indicated by dashed lines). Weight is the inverse of the variance of the maximum likelihood estimate of the log OR. Additivity of the weights is therefore only approximate. NB, presupplementation Europe subtotal allows for the common control group in Frederiksen-Prospective (P) and Frederiksen-Case-Control (CC). 95% CIs for total; other CIs are 99%. (TIF) [file pmed.1001177.s005.tif]
