# Supplementary material for: Homocysteine and Coronary Heart Disease: Meta-analysis of MTHFR Case-Control Studies, Avoiding Publication Bias
Source: PLoS Med. 2012 Feb 21;9(2):e1001177. doi: 10.1371/journal.pmed.1001177 (PMC3283559; doi:10.1371/journal.pmed.1001177)
Supplement: Text S1 — Webmaterial for homocysteine and coronary heart disease: meta-analysis of MTHFR case-control studies, avoiding publication bias. (PDF) [file pmed.1001177.s006.pdf]

Page 1: Contents list. (Click on an underlined item to jump to it)

P2: Appendix S1. Categorisation of probable folate status of each survey or study by region and year conducted (before or after nationwide folate supplementation).

P3: Appendix S2. 19 unpublished *MTHFR* datasets: calculation of the -0.1% bias in a meta-analysis of CHD TT vs CC OR arising from non-publication.

P4: Table S1. Data and CHD odds ratio (OR, TT vs CC genotype) in each of 19 unpublished datasets, based on only the homozygote genotype frequencies.

P5: Table S2. Data and CHD odds ratio (OR, TT vs CC genotype) in each of 86 published datasets, based on only the homozygote genotype frequencies.

P6: Table S3. Results for each of 70 biochemical studies of the effects of *MTHFR* genotype on homocysteine in a total of 68,369 disease-free individuals.

P7: Table S4. Results for each of 81 population-based surveys, subdivided by probable population folate status category, of serum folate in a total of 200,103 individuals.

P8: Table S5. Published randomized controlled trials that assessed the effects of folic acid on major coronary events.

P9: Table S6. Data and CHD odds ratio (OR, TT vs CC genotype) in each of the large published or unpublished Japanese datasets.

Pp 10-27: Supporting table references to each separate study.

Appendix S1. Categorisation of the probable folate status of each study by region & year conducted (before or after nationwide folate supplementation)

Legislation in 1996 permitted or mandated folic acid fortification in the United States, Canada, Australia, New Zealand, the United Kingdom and some other European countries. Changes soon followed that appreciably affected population-wide mean folate levels, but elsewhere in Europe (including Russia) the sale of manufactured foods with added folic acid remained forbidden (UK DH Scientific Advisory Committee: Folate and disease prevention. London, HMSO, 2006).

Hence, as a surrogate measurement of folate status, “probable folate status” categories were devised, based only on geographic region and calendar year of blood collection. Studies in Asia were classified as “Asia (no supplementation)”. Studies in Scandinavia, the Netherlands and Russia were classified as “Europe (pre-supplementation)” at all times, whereas studies carried out elsewhere in Europe were classified as “Europe (pre-supplementation)” before 1996 and as “Europe (post-supplementation)” from 1996 onwards. Studies carried out in North America, Australia and New Zealand were classified as “US & ANZ (pre-supplementation)” before 1996 and as “US & ANZ (post-supplementation)” from 1996 onwards.

Population mean folate levels for these 5 categories of study were estimated from a meta-analysis of all general population surveys of folate status involving more than 100 disease-free participants that were published before 2009, including not only population-based surveys of healthy individuals but also control participants in case-control studies or in randomized trials of healthy volunteers (identified using MEDLINE with search criteria folic acid, folate and B-vitamins). From 81 such population surveys, involving a total of 200,103 individuals, mean serum folate concentrations were estimated for these 5 probable folate status categories (Figure 1 and Table S1 in Text S1)

Appendix S2. 19 unpublished *MTHFR* datasets by study panel size, and calculation of the -0.1% OR bias arising from non-publication

Classification of unpublished datasets by genotyping panel size; GW=genome-wide

Dataset, ordered by number of cases	*Genotyping platform*	*Number of SNPs*	*Number of genes*	*Panel size [a] (large/small)*
ISIS	Taqman	67	24	small
deCODE	HumanHap300	300K	GW	large
PROCARDIS	HumanHap 610 Quad	50K	GW	large
PROMIS	HumanHap 660 Quad	660K	GW	large
OACIS [b]	Taqman	–	–	Large [b]
INTERHEART	GoldenGate	1536	103	small
LOLIPOP	HumanHap 610 Quad	610K	GW	large
HPS	HumanHap 610 Quad	610K	GW	large
BHF-FHS	HumanCVD	50K	2100	large
Blood-omics (Germany)	HumanCVD	50K	2100	large
Blood-omics (NL)	HumanCVD	50K	2100	large
EPIC-NL	HumanCVD	50K	2100	large
UCP	HumanCVD	50K	2100	large
Ottawa Heart Study	Affymetrix 500K / 6.0	>500K	GW	large
PennCath	HumanCVD	50K	2100	large
Yamada, GAG-u	Luminex	112	71	small
Yamada, Aichi	Luminex	300	Not spec.	small
MONICA-KORA	HumanCVD	50K	2100	large
ADVANCE	Illumina 550k v3	>500K	Not spec.	large

[a] Large panel size: at least 50K SNPs genotyped (so data-dependent publication would have required an extreme p-value, eg 10^{-6} to 10^{-8}; small panel size: <2000 SNPs genotyped (so publication could well have been triggered by a less extreme p-value, eg 10^{-3}).
[b] OACIS: Investigators were approached because they had done a 92K GWAS (on 94 MI cases, 658 controls). *MTHFR* C677T was not in that 92K panel, and was genotyped specifically for this collaboration on the much larger number of participants (3794 cases, 3479 controls) recruited into OACIS by March 2010. Although OACIS and its genotyping methods have been published, it has no *MTHFR*-related publication bias. Hence, it is grouped with the large-panel unpublished datasets that have little such bias (see below).

Size of negative bias arising from non-publication of any *MTHFR* result
If the true log OR for the *MTHFR* polymorphism is no more than about 0.08 (the value suggested by meta-analyses of prospective studies of homocysteine: see main text) then the bias in the observed log OR in a particular dataset arising from the fact that the *MTHFR* result had not been published can be shown to be less than 0.003, which would correspond to a negligibly small change (about 0.3%) in the OR. (The bias would be less than 0.003 even for small-panel studies of a limited number of polymorphisms that would have published their *MTHFR* results only if $2p<10^{-3}$ [for p calculated without allowance for multiple hypothesis testing], and it would be less than 0.0002 for large-panel studies that would have published only if $2p<10^{-6}$, so on average over all studies it would be about 0.1%).

Calculation of bias from non-publication: For a standard normal deviate d with probability P of being less than some fixed value k, it can be shown that if d is less than k then the expectation of d is $-1/(P\sqrt{2\pi})\exp(-0.5k^2)$, and that if P is close to 1 then $1/(P\sqrt{2\pi})$ is 0.4, so the expectation of d is $-0.4\exp(-0.5k^2)$.

Let β, with standard error s, denote the observed log OR from one particular study; let $z=\beta/s$; let b denote the true (but unknown) log OR, and consider the case b=0.08. (Smaller values of b would yield even smaller biases from non-publication.) If that study would have published its results on *MTHFR* only if z had been suitably large, ie, if z>X for some cut-off X, then (as long as X>b/s) the expected bias in β that arises because the result remained unpublished is $-0.4\ s\ \exp(-[X - b/s]^2/2)$.

For studies of only a small panel of polymorphisms, X might be 3.3 (corresponding to 2p<0.001), which even for the biggest of the small-panel datasets (ISIS, with s=0.08) would give a negative bias of less than 0.003. If X was 4.9 (corresponding to $2p<10^{-6}$, as used by PROCARDIS), which could arise in large-panel datasets, then even for the biggest such dataset (deCODE, with s=0.05) the bias would be less than 0.0002.

Study (country or region)	Study folate status[a]	Cases CC	Cases CT	Cases TT	Cases Odds, TT/CC	Controls CC	Controls CT	Controls TT	Controls Odds, TT/CC	Odds ratio, OR (99%CI)[b]	Weight[c]	Weight x log OR
**Large genotyping panels (>50k SNPs)**												
OACIS[d] Yokohama (Japan)[1]	1	1359	1785	650	0.478	1272	1648	559	0.439	1.09 (0.91, 1.30)	206.2	17.5
PROMIS (Pakistan)[2]	1	3383	1200	123	0.036	3262	1130	127	0.039	0.96 (0.70, 1.33)	64.0	-2.4
Blood-omics (Netherlands)[3]	2	644	656	162	0.252	567	533	122	0.215	1.17 (0.83, 1.65)	56.5	8.8
EPIC-NL (Netherlands)[4]	2	544	495	130	0.239	806	671	167	0.207	1.15 (0.83, 1.61)	59.7	8.5
MONICA-KORA [e] (Germany)[5]	2	122	119	34	0.279	597	642	174	0.291	0.96 (0.55, 1.65)	22.2	-1.0
UCP, Utrecht (Netherlands)[6]	2	535	474	119	0.222	409	372	117	0.286	0.78 (0.53, 1.13)	47.0	-11.8
PROCARDIS[f] (Sweden)[7]	2	347	251	56	0.161	350	250	69	0.197	0.82 (0.47, 1.43)	21.4	-4.3
LOLIPOP (UK South Asians)[8]	3	1830	810	101	0.055	2555	1045	96	0.038	1.30 (0.88, 1.91)	44.4	11.6
BHF-FHS (UK)[9]	3	916	943	242	0.264	1105	1027	294	0.266	0.99 (0.77, 1.28)	104.9	-0.7
Blood-omics (Germany)[10]	3	806	837	266	0.330	799	903	230	0.288	1.15 (0.88, 1.50)	94.3	12.9
deCODE (Iceland)[11]	3	2682	2886	761	0.284	11790	12145	3245	0.275	1.03 (0.92, 1.16)	480.8	14.6
HPS (UK)[12]	3	1171	1210	320	0.273	1280	1270	335	0.262	1.04 (0.83, 1.31)	129.1	5.6
PROCARDIS (UK)[7]	3	1884	1846	474	0.252	1377	1251	363	0.264			
PROCARDIS (Germany)[7]	3	151	168	41	0.272	131	142	41	0.313			
PROCARDIS (Italy)[7]	3	166	242	90	0.542	120	204	79	0.658			
PROCARDIS[f] (stratified for UK/Germany/Italy)[7]	3	2201	2256	605	0.275	1628	1597	483	0.297	0.93 (0.77, 1.13)	177.8	-12.9
Ottawa Heart Study (Canada)[13]	5	543	482	93	0.171	514	492	65	0.126	1.35 (0.87, 2.12)	33.4	10.1
PennCath (USA)[14]	5	416	465	146	0.351	200	210	79	0.395	0.89 (0.58, 1.36)	37.2	-4.4
ADVANCE (USA)[15]	5	110	127	41	0.373	135	131	46	0.341	1.09 (0.57, 2.09)	16.0	1.4
**Small genotyping panels (<2000 SNPs)**												
Yamada[g], GAG-U (Japan)[16]	1	293	411	167	0.570	997	1509	677	0.679	0.84 (0.63, 1.11)	84.2	-14.7
Yamada[g], Aichi (Japan)[17]	1	148	208	74	0.500	158	208	64	0.405	1.23 (0.73, 2.10)	23.7	5.0
INTERHEART-Middle East (Arab countries/Iran)[18]	1	486	372	85	0.175	612	438	85	0.139	1.26 (0.82, 1.93)	36.7	8.5
INTERHEART-South Asia (India/PK/BD/Nepal)[18]	1	909	307	32	0.035	1014	295	34	0.034	1.05 (0.55, 2.00)	15.9	0.8
ISIS[h] (UK)[19]	2	2981	2958	731	0.245	1168	1099	291	0.249	0.98 (0.81, 1.20)	166.8	-2.6
INTERHEART-Northern Europe (Scandinavia)[18]	2	77	75	12	0.156	179	134	32	0.179	0.87 (0.34, 2.23)	7.5	-1.0
INTERHEART-Southern Europe (Italy/Spain)[18]	3	501	486	139	0.277	495	483	138	0.279	1.00 (0.70, 1.41)	54.2	-0.3
INTERHEART-US & ANZ (Australia/Canada/USA)[18]	5	110	132	27	0.245	126	143	39	0.310	0.79 (0.38, 1.64)	12.5	-2.9

Table S1: *MTHFR* C677T genotypes in cases and controls and TT/CC CHD odds ratio (OR) in 19 unpublished datasets (with 5 parts of INTERHEART and 4 parts of PROCARDIS given separately) classified by genotyping panel size

[a] 1 = Low folate, Asia, no supplementation; 2 = Low folate, Europe, pre-supplementation; 3 = Mid folate, Europe, post-supplementation; 4 = Mid folate, US & ANZ, pre-supplementation; 5 = High folate, US & ANZ, post-supplementation

[b] OR = odds ratio = (odds of TT/CC in cases) / (odds of TT/CC in controls)

[c] Weight = inverse of (variance of log OR). Note that in PROMIS, PROCARDIS and LOLIPOP population-adjusted odds ratios were provided.

[d] OACIS: Investigator was approached because they had done a 92K GWAS (94 MI cases and 658 controls);[20] *MTHF*R C677T was not available from their panel but in response to our request it was genotyped specifically for this collaboration on the much larger number of participants (3794 cases and 3479 controls) that had been recruited into this ongoing study by March 2010. OACIS and the genotyping methods are described in the cited reference.[1] The OACIS dataset is grouped with large panels because there was no publication bias.

[e] Data from a case-cohort study analysed as case-control. If analysed as case-cohort using logistic regression the odds ratio and 99% CI are 0.94 (0.54, 1.64)

[f] PROCARDIS analyses are stratified by country, and allow for first-degree relatedness of many cases; to 2dp, this allowance does not change the OR.

[g] Yamada, GAG-U: Unpublished data (U) on those recruited 3/2005-2008 in a study in Gifu, Aomori and Gunma (GAG) prefectures. *MTHFR* results from earlier recruitment have been published and the genotyping methods remained the same.[16 21] Yamada, Aichi: Unpublished data from the screening GWA phase of a published study in the Aichi prefecture.[17]

[h] ISIS: Genotyping of *MTHFR* rs1801133 in ISIS was done along with 66 other SNPs in 24 candidate genes (population and genotyping methods described in cited reference).

Study (Country)	Year bled	Study folate status[a]	Cases CC	Cases CT	Cases TT	Cases Odds, TT/CC	Controls CC	Controls CT	Controls TT	Controls Odds, TT/CC	Odds ratio, OR (99%CI)[b]	Weight [c]	Weight[c] x log OR
Abu-Amero (Saudi Arabia)[1]	2003	1	350	175	20	0.057	451	161	13	0.029	1.98 (0.79, 4.98)	7.8	5.4
Chao (Taiwan)[2]	1997	1	60	47	9	0.150	41	25	10	0.244	0.61 (0.17, 2.25)	3.9	-1.9
Chen (Taiwan)[3]	2003	1	33	23	4	0.121	62	42	5	0.081	1.52 (0.23, 9.87)	1.9	0.8
Gulec (Turkey)[4]	1999	1	42	39	15	0.357	60	35	5	0.083	3.85 (1.09,13.56)	4.2	5.7
Hong (Korea)[5]	1999	1	40	74	26	0.650	37	78	25	0.676	0.96 (0.38, 2.44)	7.7	-0.3
Hsu (China)[6]	1999	1	120	85	13	0.108	125	78	15	0.120	0.90 (0.32, 2.52)	6.3	-0.6
Ilhan (Turkey)[7]	2008	1	52	44	4	0.077	72	26	2	0.028	2.72 (0.31,23.82)	1.4	1.4
Inbal (Israel)[8]	1996	1	29	56	27	0.931	85	82	20	0.235	4.06 (1.59,10.36)	7.6	10.7
Izumi (Japan)[9]	1995	1	90	110	50	0.556	74	102	25	0.338	1.62 (0.78, 3.36)	12.5	6.1
Jang (Korea)[10]	2000	1	49	77	23	0.469	67	115	48	0.716	0.66 (0.30, 1.47)	10.5	-4.3
Kawashiri (Japan)[11]	2000	1	33	51	15	0.455	48	45	7	0.146	2.98 (0.86,10.27)	4.3	4.7
Kihara (Japan)[12]	1997	1	29	57	13	0.448	51	65	16	0.314	1.43 (0.46, 4.49)	5.1	1.8
Kim (Korea)[13]	2000	1	30	41	14	0.467	56	68	28	0.500	0.93 (0.34, 2.59)	6.4	-0.4
Kou (Taiwan)[14]	2000	1	29	19	6	0.207	35	18	2	0.057	3.23 (0.47,22.07)	1.8	2.1
Mager (Israel)[15]	1999	1	16	32	19	1.188	130	139	44	0.338	3.94 (1.39,11.13)	6.2	8.4
Morita (Japan)[16]	1996	1	117	188	57	0.487	338	361	79	0.234	2.18 (1.25, 3.79)	21.8	17.0
Nakai (Japan)[17]	1997	1	93	95	42	0.452	81	96	21	0.259	1.71 (0.80, 3.67)	11.4	6.1
Ou (Japan)[18]	1997	1	69	84	61	0.884	110	158	42	0.382	2.29 (1.21, 4.34)	16.3	13.5
Shioji (Japan)[19]	2003	1	193	250	88	0.456	663	878	305	0.460	0.99 (0.68, 1.44)	47.0	-0.4
Tokgozoglu (Turkey)[20]	1998	1	69	71	11	0.159	47	39	5	0.106	1.47 (0.36, 5.98)	3.4	1.3
Yamada 2006 (Japan) [21]	2004	1	375	570	247	0.659	804	1134	353	0.439	1.51 (1.15, 1.98)	90.5	37.2
Zhang (China)[22]	2000	1	32	33	8	0.250	37	47	16	0.432	0.59 (0.17, 2.02)	4.4	-2.3
Zheng (China)[23]	1999	1	41	29	2	0.049	62	45	15	0.242	0.30 (0.07, 1.21)	3.4	-4.1
Abbate (Italy)[24]	1995	2	19	41	24	1.263	26	48	32	1.231	1.03 (0.36, 2.90)	6.2	0.2
Ardissino (Italy)[25]	1995	2	68	97	35	0.515	60	102	38	0.633	0.81 (0.38, 1.73)	11.7	-2.4
Fowkes (UK)[26]	1989	2	59	66	12	0.203	132	147	21	0.159	1.29 (0.45, 3.65)	6.1	1.5
Frederiksen-CC[d] (Denmark)[27]	2002	2	1048	867	210	0.200	3675	3258	692	0.188	1.06 (0.85, 1.33)	131.5	8.3
Frederiksen-P[d] (Denmark)[27]	2002	2	506	443	94	0.186	3675	3258	692	0.188	0.99 (0.73, 1.34)	70.3	-0.9
Gallagher (Ireland)[28]	1991	2	44	48	19	0.432	53	45	7	0.132	3.01 (0.97, 9.35)	5.2	5.7
Kluijtmans (Netherlands)[29]	1990	2	337	328	70	0.208	617	527	106	0.172	1.21 (0.78, 1.88)	34.3	6.6
Kostulas (Sweden)[30]	1997	2	63	50	13	0.206	63	50	13	0.206	1.00 (0.33, 3.03)	5.4	0.0
Meleady (Ireland)[31]	1991	2	313	307	91	0.291	352	314	81	0.230	1.26 (0.81, 1.96)	34.2	8.0
Roest (Netherlands)[32]	1977	2	109	95	21	0.193	209	248	58	0.278	0.71 (0.35, 1.41)	14.0	-4.9
Spiridonova (Russia)[33]	2002	2	44	41	9	0.205	64	48	10	0.156	1.31 (0.36, 4.79)	4.0	1.1
Tanis (Netherlands)[34]	1999	2	78	81	22	0.282	280	262	59	0.211	1.36 (0.64, 2.89)	11.6	3.5
Thogersen (Sweden)[35]	1988	2	32	32	5	0.156	71	51	7	0.099	1.62 (0.30, 8.66)	2.4	1.1
Verhoef (Netherlands)[36]	1993	2	59	59	13	0.220	45	48	7	0.156	1.40 (0.39, 4.99)	4.1	1.4
Verhoeff (Netherlands)[37]	1997	2	115	83	19	0.165	129	105	38	0.295	0.57 (0.27, 1.23)	11.5	-6.4
Adams (UK)[38]	1996	3	133	145	32	0.241	96	97	29	0.302	0.80 (0.38, 1.68)	11.9	-2.7
Araujo (Portugal)[39]	1999	3	74	103	22	0.297	10	8	2	0.200	1.43 (0.22, 9.48)	1.9	0.7
Benes (Czech Rep.)[40]	1998	3	264	309	68	0.258	234	265	59	0.252	1.02 (0.61, 1.71)	25.2	0.5
Brulhart (France)[41]	1997	3	86	84	23	0.267	188	195	73	0.388	0.70 (0.36, 1.37)	14.8	-5.3
Chambers-Asian (UK)[42]	1999	3	160	61	3	0.019	279	90	12	0.043	0.49 (0.12, 2.01)	3.3	-2.4
Chambers-European (UK)[42]	1999	3	108	91	31	0.287	188	195	41	0.218	1.32 (0.66, 2.66)	13.6	3.8
Fernandez-Arcas (Spain)[43]	1998	3	104	107	61	0.587	82	92	39	0.476	1.23 (0.64, 2.35)	15.9	3.3
Ferrer (Portugal)[44]	1998	3	14	19	7	0.500	26	16	8	0.308	1.62 (0.33, 7.99)	2.6	1.3
Ferrer-Antunes (Portugal)[45]	1998	3	54	59	14	0.259	71	51	5	0.070	3.37 (0.95,11.97)	4.1	5.0
Gardemann (Germany)[46]	1996	3	891	805	197	0.221	88	82	15	0.170	1.27 (0.64, 2.55)	13.8	3.3
Gemmati (Italy)[47]	1999	3	32	81	47	1.469	68	102	30	0.441	3.22 (1.47, 7.05)	10.8	12.6
Girelli (Italy)[48]	1997	3	146	217	70	0.479	75	105	42	0.560	0.86 (0.46, 1.60)	17.0	-2.6
Gorqcy (Poland)[49]	1999	3	46	45	9	0.196	39	50	11	0.282	0.70 (0.19, 2.50)	4.1	-1.5
Kadziela (Poland)[50]	2003	3	58	51	11	0.190	46	49	11	0.239	0.79 (0.24, 2.67)	4.5	-1.0
Kolling (Germany)[51]	2004	3	915	955	251	0.274	266	283	68	0.256	1.07 (0.73, 1.59)	43.5	3.0
Kozich (Czech Rep.)[52]	1999	3	123	117	38	0.309	245	287	59	0.241	1.29 (0.69, 2.39)	17.4	4.4
Malik (UK)[53]	1998	3	107	134	26	0.243	106	110	17	0.160	1.50 (0.64, 3.56)	9.0	3.7
Mannucci (Italy)[25]	2000	3	371	547	292	0.787	356	607	247	0.694	1.13 (0.85, 1.52)	77.3	9.7
Meisel (Germany)[54]	1996	3	458	442	81	0.177	443	442	96	0.217	0.82 (0.53, 1.25)	37.0	-7.5
Pinto (Spain)[55]	2004	3	31	34	11	0.355	39	43	13	0.333	1.06 (0.31, 3.61)	4.5	0.3
Raslova (Slovakia)[56]	1999	3	22	29	7	0.318	59	46	6	0.102	3.45 (0.66,18.13)	2.4	3.0
Reinhardt (Germany)[57]	1996	3	91	66	23	0.253	49	46	9	0.184	1.36 (0.47, 3.94)	5.9	1.8
Rossi (Italy)[58]	2000	3	42	53	29	0.690	23	33	17	0.739	0.93 (0.33, 2.62)	6.3	-0.4
Rothenbacher (Germany)[59]	1996	3	155	126	31	0.200	219	210	50	0.228	0.88 (0.46, 1.67)	16.1	-2.1
Sarecka-Hujar (Poland)[60]	2002	3	75	85	17	0.227	103	89	10	0.097	2.31 (0.79, 6.73)	5.8	4.9
Szczeklik (Poland)[61]	2000	3	105	39	17	0.162	201	94	15	0.075	2.25 (0.83, 6.11)	6.7	5.4
Tobin (UK)[62]	1998	3	236	246	65	0.275	214	230	61	0.285	0.97 (0.57, 1.63)	24.6	-0.8
Todesco (Germany)[63]	1999	3	30	34	11	0.367	103	93	28	0.272	1.36 (0.45, 4.09)	5.5	1.7
Zuntar (Croatia)[64]	2003	3	114	111	22	0.193	134	146	18	0.134	1.43 (0.60, 3.46)	8.6	3.1
Brugada (USA)[65]	1995	4	76	69	10	0.132	70	73	12	0.171	0.77 (0.24, 2.50)	4.8	-1.3
Dilley (USA)[66]	1995	4	91	17	2	0.022	153	28	4	0.026	0.84 (0.09, 7.63)	1.4	-0.2
Folsom (USA)[67]	1988	4	148	109	17	0.115	271	187	47	0.173	0.68 (0.33, 1.41)	12.5	-4.9
Hopkins (USA)[68]	1995	4	124	82	24	0.194	69	62	16	0.232	0.83 (0.33, 2.11)	7.7	-1.4
Ma (USA)[69]	1982	4	136	124	33	0.243	135	116	39	0.289	0.84 (0.42, 1.66)	14.3	-2.5
Schmitz (USA)[70]	1982	4	95	66	29	0.305	71	90	27	0.380	0.80 (0.36, 1.79)	10.4	-2.3
Schwartz (USA)[71]	1993	4	28	34	7	0.250	154	141	43	0.279	0.90 (0.28, 2.83)	5.0	-0.5
Silberberg (Australia)[72]	1993	4	139	101	34	0.245	50	51	11	0.220	1.11 (0.42, 2.93)	7.0	0.7
Verhoef (USA)[73]	1994	4	230	209	61	0.265	228	200	72	0.316	0.84 (0.51, 1.40)	25.8	-4.5
Wilcken (Australia)[74]	1995	4	186	217	53	0.285	88	113	24	0.273	1.04 (0.51, 2.13)	13.1	0.6
Anderson (USA)[75]	1997	5	241	212	57	0.237	73	73	22	0.301	0.78 (0.37, 1.66)	11.6	-2.9
Brilakis (USA)[76]	2002	5	117	123	31	0.265	53	52	17	0.321	0.82 (0.33, 2.03)	8.2	-1.6
Christensen (Canada)[77]	1996	5	62	68	22	0.355	47	61	13	0.277	1.28 (0.46, 3.52)	6.5	1.6
Hanson (USA)[78]	2000	5	324	364	84	0.259	130	158	41	0.315	0.82 (0.46, 1.45)	20.4	-4.1
Malinow (USA)[79]	1996	5	40	83	17	0.425	49	45	8	0.163	2.49 (0.78, 7.98)	4.9	4.5
Payne (USA)[80]	2001	5	20	20	5	0.250	17	6	1	0.059	3.24 (0.33,31.49)	1.3	1.5
Topol (USA)[81]	2000	5	151	115	47	0.311	191	172	46	0.241	1.29 (0.71, 2.37)	18.2	4.7
Tsai (USA)[82]	1998	5	159	177	40	0.252	35	35	12	0.343	0.72 (0.26, 2.01)	6.4	-2.1
van Bockxmeer (Australia)[83]	1996	5	234	226	58	0.248	67	57	16	0.239	1.04 (0.46, 2.32)	10.3	0.4

Table S2: *MTHFR* C677T genotypes in cases and controls and TT/CC CHD odds ratio (OR) in 86 published CHD case-control studies (NB Many studies did not measure homocysteine)

[a] 1 = Low folate, Asia, no supplementation; 2 = Low folate, Europe, pre-supplementation; 3 = Mid folate, Europe, post-supplementation; 4 = Mid folate, US & ANZ, pre-supplementation; 5 = High folate, US & ANZ, post-supplementation

[b] OR = (odds of TT/CC in cases)/(odds of TT/CC in controls)

[c] Weight = inverse of (variance of log OR)

[d] Combined result for Frederiksen (CC&P): 1.04 (0.86,1.26), weight = 175.2, weight x (log OR) = 6.7

Study (Country)	Year bled	Folate status[a]	CHD study (Y=Yes)	No. of disease-free people [b]			Geometric mean homocysteine μmol/L		
				CC	CT	TT	CC	CT	TT
Chao (Taiwan)[1]	1997	1	Y	101	72	19	9.6	10.7	12.4
Nakai (Japan)[2]	1997	1	Y	81	96	21	7.9	8.2	10.6
Ou (Japan)[3]	1997	1	Y	110	158	42	10.2	10.8	14.6
Tokgozoglu (Turkey)[4]	1998	1	Y	24	21	3	12.2	16.2	33.0
Friedman (Israel)[5]	1999	1	N	150	172	55	8.7	8.7	10.4
Ho (China)[6]	2000	1	N	280	186	69	7.5	7.7	8.5
Jang (Korea)[7]	2000	1	Y	67	115	48	8.1	8.2	12.4
Kawashiri (Japan)[8]	2000	1	Y	33	51	15	9.3	10.2	19.5
Kim (Korea)[9]	2000	1	Y	30	40	15	11.7	12.1	12.9
Saw (Singapore)[10]	2000	1	N	273	165	39	9.7	10.0	12.3
Somekawa (Japan)[11]	2001	1	N	96	91	30	9.6	10.3	10.6
Inamoto (Japan)[12]	2002	1	N	1189	1539	520	11.0	11.2	13.3
Jee (South Korea)[13]	2002	1	N	135	184	76	8.5	8.6	11.8
Yingdong (China)[14]	2002	1	N	21	16	5	10.9	13.2	20.5
Ilhan (Turkey)[15]	2008	1	Y	72	26	2	12.4	12.3	11.2
Thogersen (Sweden)[16]	1988	2	Y	67	49	7	11.8	11.1	11.4
Kluijtmans (Netherlands)[17]	1990	2	Y	231	233	51	12.6	13.4	15.4
Meleady (Ireland)[18]	1991	2	Y	340	305	78	9.3	9.6	12.2
Devlin (UK)[19]	1993	2	N	488	432	121	12.3	12.8	14.7
Verhoef (Netherlands)[20]	1993	2	Y	45	48	7	10.9	12.1	17.4
Verhoeff (Netherlands)[21]	1997	2	Y	129	105	38	12.7	13.2	17.6
Tanis (Netherlands)[22]	1999	2	Y	296	272	60	11.3	12.2	14.0
Hustad-1 (Norway)[23]	2000	2	N	204	182	37	8.6	9.1	11.5
Silaste (Finland)[24]	2001	2	N	19	13	5	7.8	7.8	8.8
deBree (Netherlands)[25]	2001	2	N	983	907	206	12.9	13.6	17.1
Bathum (Denmark)[26]	2002	2	N	556	475	102	6.9	7.5	10.5
Frederiksen-P (Denmark)[27]	2002	2	Y	1804	1600	315	10.9	11.0	13.8
Husemoen (Denmark)[28]	2003	2	N	1361	1181	246	7.8	8.2	9.8
Hustad-2 (Norway)[29]	2007	2	N	5452	4299	850	9.9	10.4	11.2
Dekou (UK)[30]	1996	3	N	345	337	82	11.1	11.4	12.3
Meisel (Germany)[31]	1996	3	Y	418	410	93	9.7	9.7	10.0
Rothenbacher (Germany)[32]	1996	3	Y	219	210	50	8.0	8.1	9.8
Chango (France)[33]	1997	3	N	29	25	12	8.5	8.8	10.1
Chango(S) (France)[34]	1997	3	N	117	125	49	9.3	9.0	10.7
Girelli (Italy)[35]	1997	3	Y	40	74	23	12.9	13.5	13.6
Ferrer (Portugal)[36]	1998	3	Y	24	12	6	7.3	9.1	7.9
Thuillier (France)[37]	1998	3	N	27	32	10	7.6	8.2	8.5
Zittoun (France)[38]	1998	3	N	20	20	6	10.9	11.5	19.0
Chambers-Asian (UK)[39]	1999	3	Y	278	89	12	11.0	10.4	10.4
Chambers-European (UK)[39]	1999	3	Y	187	194	41	10.1	10.1	11.6
Gemmati (Italy)[40]	1999	3	Y	70	98	32	6.9	7.5	10.7
Kozich (Czech Rep.)[41]	1999	3	Y	245	287	59	9.2	9.7	11.5
Todesco (Germany)[42]	1999	3	Y	102	92	26	10.1	10.2	10.9
D'Angelo (Italy)[43]	2000	3	N	45	98	39	9.3	10.4	16.6
Passaro (Italy)[44]	2000	3	N	28	72	20	9.1	10.3	12.4
Szczeklik (Poland)[45]	2000	3	Y	199	95	15	10.8	11.1	9.8
Cappuccio (UK)[46]	2001	3	N	916	391	64	10.0	10.4	11.3
Fohr (Germany)[47]	2001	3	N	64	75	21	8.2	8.2	8.1
Madonna (Italy)[48]	2001	3	N	102	105	45	8.8	9.0	13.6
Pullin (UK)[49]	2001	3	N	42	42	42	8.3	8.8	11.8
Castro (Portugal)[50]	2002	3	N	51	54	12	8.3	9.2	9.8
Litynski (Switzerland)[51]	2002	3	N	20	.	20	11.9	.	15.9
Reyes-Engel (Spain)[52]	2002	3	N	249	413	119	9.8	10.1	11.9
Kolling (Germany)[53]	2004	3	Y	266	283	68	11.0	11.2	12.4
Ma (USA)[54]	1982	4	Y	136	116	39	9.8	10.3	11.1
Schmitz (USA)[55]	1982	4	Y	28	25	6	9.0	9.8	8.7
Folsom (USA)[56]	1988	4	Y	266	186	46	8.6	9.0	11.3
Jacques (USA)[57]	1993	4	N	149	152	149	8.9	9.1	9.8
Schwartz (USA)[58]	1993	4	Y	167	159	47	10.5	10.2	12.2
Silberberg (Australia)[59]	1993	4	Y	30	32	5	11.3	12.2	12.7
Zee (USA)[60]	1994	4	N	11229	10966	2773	10.2	10.5	11.5
Hopkins (USA)[61]	1995	4	Y	69	62	16	9.5	10.2	9.9
Christensen (Canada)[62]	1996	5	Y	47	61	13	8.0	8.9	9.2
Malinow (USA)[63]	1996	5	Y	47	47	11	9.2	8.2	9.2
Anderson (USA)[64]	1997	5	Y	63	61	17	14.0	14.7	13.3
Eikelboom (Australia)[65]	1997	5	N	84	98	23	10.2	11.5	11.9
Tsai (USA)[66]	1998	5	Y	29	34	12	8.0	8.7	9.0
Hanson (USA)[67]	2000	5	Y	512	585	141	8.7	8.9	9.6
Caudill (USA)[68]	2001	5	N	75	50	9	5.3	5.4	4.0
Guinotte (USA)[69]	2002	5	N	14	12	17	5.2	5.8	5.3

Table S3: Effects of *MTHFR* C677T genotype on homocysteine in 70 biochemical studies of 68 369 disease-free individuals: 37 CHD case-control studies of *MTHFR* and 33 other studies of *MTHFR* with homocysteine measured in some disease-free people.

[a] 1 = Low folate, Asia, no supplementation; 2 = Low folate, Europe, pre-supplementation; 3 = Mid folate, Europe, post-supplementation; 4 = Mid folate, US & ANZ, pre-supplementation; 5 = High folate, US & ANZ, post-supplementation

[b] Controls only, except for a few case-control studies where biochemical results were available only for cases, or only for cases and controls combined

Study (Country)	Year bled	Folate status [a]	No. of people	Mean serum folate (nmol/L)
Ronnenberg (China)[1]	1997	1	563	9.7
Wang (China)[2]	1998	1	963	10.6
Friedman (Israel)[3]	1999	1	377	8.9
Jang (South Korea)[4]	2000	1	230	11.9
Saw (Singapore)[5]	2000	1	477	13.8
Hao (China)[6]	2001	1	1836	11.1
Jee (South Korea)[7]	2002	1	395	11.2
Hultdin (Sweden)[8]	1985	2	514	8.8
vanGuelpen (Sweden)[9]	1985	2	671	7.2
Hartman (Finland)[10]	1986	2	325	9.7
Meleady (Ireland)[11]	1991	2	723	9.7
Nurk (Norway)[12]	1992	2	7031	7.1
Voutilainen (Finland)[13]	1992	2	749	10.5
Clarke (UK)[14]	1993	2	1500	11.0
Devlin (UK)[15]	1993	2	1042	11.0
deBree (Netherlands)[16]	1994	2	2051	7.4
Dierkes-1 (Germany)[17]	1995	2	198	21.0
Melse-Boonstra (Netherlands)[18]	1995	2	2435	8.3
Naurath (Belgium)[19]	1995	2	285	9.7
Woodside (Ireland)[20]	1995	2	112	9.2
Alfthan (Finland)[21]	1997	2	318	14.6
Wahlin (Sweden)[22]	1997	2	961	17.9
Brouwer (Netherlands)[23]	1998	2	144	11.0
Tanis (Netherlands)[24]	1999	2	617	8.7
Durga (Netherlands)[25]	2000	2	801	12.0
Hustad-1 (Norway)[26]	2000	2	423	15.3
Bjorkegren (Sweden)[27]	2004	2	266	10.0
Hustad-2 (Norway)[28]	2007	2	10601	17.1
Dekou (UK)[29]	1996	3	408	6.0
Trobs (Germany)[30]	1996	3	817	18.0
Brown (Ireland)[31]	1997	3	922	11.6
Chango(S) (France)[32]	1997	3	291	16.4
Girelli (Italy)[33]	1997	3	124	15.5
Dierkes-2 (Germany)[34]	1998	3	150	29.3
Friso (Italy)[35]	1998	3	267	12.9
Chambers-Asian (UK)[36]	1999	3	379	16.6
Chambers-European (UK)[36]	1999	3	418	18.1
Duthie (UK)[37]	1999	3	331	17.2
Gemmati (Italy)[38]	1999	3	200	11.5
Kozich (Czech Rep.)[39]	1999	3	591	16.1
MacMahon (UK)[40]	1999	3	137	16.2
Ravaglia (Italy)[41]	1999	3	599	13.6
Ashfield-Watt (UK)[42]	2000	3	126	17.7
D'Angelo (Italy)[43]	2000	3	182	11.9
Dierkes-3 (Germany)[44]	2000	3	336	16.8
Mayer (Czech Rep.)[45]	2000	3	543	14.0
Rauh (Germany)[46]	2000	3	172	16.5
Szczeklik (Poland)[47]	2000	3	309	13.6
Fohr (Germany)[48]	2001	3	160	15.7
Pullin (UK)[49]	2001	3	126	17.2
Ruston (UK)[50]	2001	3	1314	21.5
Wolters (Germany)[51]	2001	3	178	20.1
Castro (Portugal)[52]	2002	3	117	14.5
Hatzis (Greece)[53]	2002	3	486	17.3
Kadziela (Poland)[54]	2003	3	106	20.2
Kolling (Germany)[55]	2004	3	617	21.8
Nelson (UK)[56]	2004	3	2796	25.0
Obeid (Germany)[57]	2004	3	302	15.3
Ma (USA)[58]	1982	4	290	12.6
Verhoef (USA)[59]	1983	4	118	9.9
Folsom (USA)[60]	1988	4	480	9.8
Pfeiffer-1 (USA)[61]	1990	4	23361	12.4
Zhang (USA)[62]	1990	4	712	16.5
Dalery (Canada)[63]	1991	4	584	9.3
Jacques-1 (USA)[64]	1993	4	756	10.4
Jacques-2 (USA)[65]	1993	4	450	12.7
Schwartz (USA)[66]	1993	4	368	15.7
Hopkins (USA)[67]	1995	4	146	24.7
Jacques-3 (USA)[68]	1995	4	365	15.4
Lawrence-1 (USA)[69]	1995	4	29243	28.7
Robinson (USA)[70]	1995	4	231	17.9
Christensen (Canada)	1996	5	121	9.5
Jacques-4 (USA)[64]	1996	5	350	22.7
Malinow (USA)[71]	1996	5	105	19.5
Aronow (USA)[72]	1997	5	306	21.3
Eikelboom (Australia)[73]	1997	5	205	16.8
Lawrence-2 (USA)[69]	1998	5	67109	35.2
Caudill (USA)[74]	2001	5	134	50.0
Hickling (Australia)[75]	2001	5	936	19.5
McMahon (New Zealand)[76]	2003	5	276	22.2
Pfeiffer-2 (USA)[61]	2004	5	23345	29.4

Table S4: Population-based surveys of serum folate, subdivided by probable folate status category, as defined by country and year of blood collection (NB Many had unknown homocysteine or genotype.)

[a] 1 = Low folate, Asia, no supplementation; 2 = Low folate, Europe, pre-supplementation; 3 = Mid folate, Europe, post-supplementation; 4 = Mid folate, US & ANZ, pre-supplementation; 5 = High folate, US & ANZ, post-supplementation

Trial name	Number of participants	Number of CHD events
SU.FOL.OM3[1]	2501	104
VISP[2]	3680	170
WAFACS[2]	5442	194
VITATOPS[3]	8164	232
WENBIT[2]	3090	248
HOST[2]	2056	325
FAVORIT[a 4]	4110	488
NORVIT[2]	3749	689
HOPE-2[2]	5522	814
SEARCH[2]	12064	1550
**ALL**	**50378**	**4814**

Table S5: Published randomized controlled trials that assessed the effects of folic acid on major coronary events.

[a] Results for the FAVORIT trial, that assessed the effects of homocysteine reduction in 4110 renal transplant participants have not yet been published for CHD alone, but, preliminary results indicated no significant effect of homocysteine reduction on cardiovascular disease.

	Cases						Controls						Odds ratio,		
	CC	CT	TT	Odds, TT/CC	T allele freq.	H-W p-value	CC	CT	TT	Odds, TT/CC	T allele freq	H-W p-value	OR[a] (99% or 95% CI[c])	Weight[b]	Weight[b] x log OR
**Japanese unpublished datasets**															
OACIS, Yokohama	1359	1785	650	0.478	0.407	0.124	1272	1648	559	0.439	0.398	0.514	1.09 (0.91, 1.30)	206.2	17.5
Yamada, GAG-U	293	411	167	0.570	0.428	0.287	997	1509	677	0.679	0.450	0.017	0.84 (0.63, 1.11)	84.2	-14.7
Yamada, Aichi	148	208	74	0.500	0.414	0.950	158	208	64	0.405	0.391	0.740	1.23 (0.73, 2.10)	23.7	5.0
**Subtotal (by addition)**	**1800**	**2404**	**891**				**2427**	**3365**	**1300**				**1.02 (0.92, 1.14)**	**314.1**	**7.7**
**Japanese large published studies**															
Morita, 1996	117	188	57	0.487	0.417	0.196	338	361	79	0.234	0.334	0.223	2.08 (1.23, 3.53)	24.0	17.6
Shioji, 2003	193	250	88	0.456	0.401	0.644	663	878	305	0.460	0.403	0.619	0.99 (0.68, 1.44)	46.9	-0.4
Yamada, 2006	375	570	247	0.659	0.446	0.262	804	1134	353	0.439	0.402	0.153	1.50 (1.15, 1.96)	92.7	37.6
**Subtotal (by addition)**	**685**	**1008**	**392**				**1805**	**2373**	**737**				**1.40 (1.20, 1.63)**	**163.5**	**54.8**
Japanese comparators, from Table S3[d]							1509	1935	628	0.416					

Table S6: Details of datasets and calculations used to derive the CHD odds ratio (OR, TT vs CC *MTHFR* C677T genotype) in the large Japanese studies; p-values test genotype frequencies for Hardy-Weinberg equilibrium.

[a] In individual studies, CHD odds ratio (OR) = (odds of TT/CC in cases) / (odds of TT/CC in controls)
[b] Weight = inverse of (variance of log OR). Additivity of weights is therefore only approximate.
[c] 95% CIs are given for subtotals and totals, 99% CIs for other results
[d] There is no overlap between the controls in the large published studies in this table and the comparators (ie, the genotyped Japanese participants in Table S3 in Text S1).

REFERENCES

References for Table S1 (Unpublished datasets of *MTHFR* and CHD)

S1-1. Ozaki K, Sato H, Iida A *et al*. A functional SNP in PSMA6 confers risk of myocardial infarction in the Japanese population. Nat Genet 2006; 38(8):921-5.

S1-2. Saleheen D, Zaidi M, Rasheed A *et al*. The Pakistan Risk of Myocardial Infarction Study: a resource for the study of genetic, lifestyle and other determinants of myocardial infarction in South Asia. Eur J Epidemiol 2009; 24(6):329-38.

S1-3. Macaulay IC, Tijssen MR, Thijssen-Timmer DC *et al*. Comparative gene expression profiling of in vitro differentiated megakaryocytes and erythroblasts identifies novel activatory and inhibitory platelet membrane proteins. Blood 2007; 109(8):3260-9.

S1-4. Beulens JW, Monninkhof EM, Verschuren WM *et al*. Cohort Profile: The EPIC-NL study. Int J Epidemiol 2009.

S1-5. Wichmann HE, Gieger C, Illig T. KORA-gen--resource for population genetics, controls and a broad spectrum of disease phenotypes. Gesundheitswesen 2005; 67 Suppl 1:S26-30.

S1-6. van Wieren-de Wijer DB, Maitland-van der Zee AH, de Boer A *et al*. Interaction between the Gly460Trp alpha-adducin gene variant and diuretics on the risk of myocardial infarction. J Hypertens 2009; 27(1):61-8.

S1-7. Clarke R, Peden JF, Hopewell JC *et al*. Genetic variants associated with Lp(a) lipoprotein level and coronary disease. N Engl J Med 2009; 361(26):2518-28.

S1-8. Chambers JC, Zhao J, Terracciano CM *et al*. Genetic variation in SCN10A influences cardiac conduction. Nat Genet 2010; 42(2):149-52.

S1-9. Samani NJ, Burton P, Mangino M *et al*. A genomewide linkage study of 1,933 families affected by premature coronary artery disease: The British Heart Foundation (BHF) Family Heart Study. Am J Hum Genet 2005; 77(6):1011-20.

S1-10. Meisinger C, Prokisch H, Gieger C *et al*. A genome-wide association study identifies three loci associated with mean platelet volume. Am J Hum Genet 2009; 84(1):66-71.

S1-11. Helgadottir A, Thorleifsson G, Manolescu A *et al*. A common variant on chromosome 9p21 affects the risk of myocardial infarction. Science 2007; 316(5830):1491-3.

S1-12. Heart Protection Study Collaborative Group. MRC/BHF Heart Protection Study of cholesterol lowering with simvastatin in 20,536 high-risk individuals: a randomised placebo-controlled trial. Lancet 2002; 360(9326):7-22.

S1-13. Stewart AF, Dandona S, Chen L *et al*. Kinesin family member 6 variant Trp719Arg does not associate with angiographically defined coronary artery disease in the Ottawa Heart Genomics Study. J Am Coll Cardiol 2009; 53(16):1471-2.

S1-14. Lehrke M, Millington SC, Lefterova M *et al*. CXCL16 is a marker of inflammation, atherosclerosis, and acute coronary syndromes in humans. J Am Coll Cardiol 2007; 49(4):442-9.

S1-15. Assimes TL, Knowles JW, Basu A *et al*. Susceptibility locus for clinical and subclinical coronary artery disease at chromosome 9p21 in the multi-ethnic ADVANCE study. Hum Mol Genet 2008; 17(15):2320-8.

S1-16. Yamada Y, Fuku N, Tanaka M *et al*. Identification of CELSR1 as a susceptibility gene for ischemic stroke in Japanese individuals by a genome-wide association study. Atherosclerosis 2009; 207(1):144-9.

S1-17. Yamada Y, Izawa H, Ichihara S *et al*. Prediction of the risk of myocardial infarction from polymorphisms in candidate genes. N Engl J Med 2002; 347(24):1916-23.

S1-18. Anand SS, Xie C, Pare G *et al*. Genetic variants associated with myocardial infarction risk factors in over 8000 individuals from five ethnic groups: The INTERHEART Genetics Study. Circ Cardiovasc Genet 2009; 2(1):16-25.

S1-19. Clarke R, Xu P, Bennett D *et al*. Lymphotoxin-alpha gene and risk of myocardial infarction in 6,928 cases and 2,712 controls in the ISIS case-control study. PLoS Genet 2006; 2(7):e107.

S1-20. Ozaki K, Ohnishi Y, Iida A *et al*. Functional SNPs in the lymphotoxin-alpha gene that are associated with susceptibility to myocardial infarction. Nat Genet 2002; 32(4):650-4.

S1-21. Yamada Y, Matsuo H, Segawa T *et al*. Assessment of genetic risk for myocardial infarction. Thromb Haemost 2006; 96(2):220-7.

References for Table S2 (Primary studies *MTHFR* and CHD)

S2-1. Abu-Amero KK, Wyngaard CA, Dzimiri N. Prevalence and role of methylenetetrahydrofolate reductase 677 C-->T and 1298 A-->C polymorphisms in coronary artery disease in Arabs. Arch Pathol Lab Med 2003; 127(10):1349-52.

S2-2. Chao CL, Tsai HH, Lee CM *et al*. The graded effect of hyperhomocysteinemia on the severity and extent of coronary atherosclerosis. Atherosclerosis 1999; 147(2):379-86.

S2-3. Chen TY, Chen JH, Tsao CJ. Methylenetetrahydrofolate reductase gene polymorphism and coronary artery disease in Taiwan Chinese. Haematologica 2000; 85(4):445-6.

S2-4. Gulec S, Aras O, Akar E *et al*. Methylenetetrahydrofolate reductase gene polymorphism and risk of premature myocardial infarction. Clin Cardiol 2001; 24(4):281-4.

S2-5. Hong SH, Song J, Kim JQ. Genetic variation of the methylenetetrahydrofolate reductase and cystathionine beta-synthase genes in Korean patients with coronary artery disease and a new polymorphism in intron 7. Mol Cell Probes 2001; 15(2):119-23.

S2-6. Hsu LA, Ko YL, Wang SM *et al*. The C677T mutation of the methylenetetrahydrofolate reductase gene is not associated with the risk of coronary artery disease or venous thrombosis among Chinese in Taiwan. Hum Hered 2001; 51(1-2):41-5.

S2-7. Ilhan N, Kucuksu M, Kaman D, Ilhan N, Ozbay Y. The 677 C/T MTHFR polymorphism is associated with essential hypertension, coronary artery disease, and higher homocysteine levels. Arch Med Res 2008; 39(1):125-30.

S2-8. Inbal A, Freimark D, Modan B *et al*. Synergistic effects of prothrombotic polymorphisms and atherogenic factors on the risk of myocardial infarction in young males. Blood 1999; 93(7):2186-90.

S2-9. Izumi M, Iwai N, Ohmichi N, Nakamura Y, Shimoike H, Kinoshita M. Molecular variant of 5,10-methylenetetrahydrofolate reductase is a risk factor of ischemic heart disease in the Japanese population. Atherosclerosis 1996; 121(2):293-4.

S2-10. Jang Y, Park HY, Lee JH, Ryu HJ, Kim JY, Kim OY. A polymorphism of the methylenetetrahydrofolate reductase and methionine synthase gene in CAD patients: association with plasma folate, vitamin B12 and homocysteine. Nutrition Research 2002; 22(9):965-76.

S2-11. Kawashiri M, Kajinami K, Nohara A *et al*. Effect of common methylenetetrahydrofolate reductase gene mutation on coronary artery disease in familial hypercholesterolemia. Am J Cardiol 2000; 86(8):840-5.

S2-12. Kihara T, Abe S, Saigo M *et al*. Methelenetetrahydrofoalte Reductase Gene Polymorphism and Premature Myocardial Infarction. 96. 1997.

S2-13. Kim CH, Hwang KY, Choi TM, Shin WY, Hong SY. The methylenetetrahydrofolate reductase gene polymorphism in Koreans with coronary artery disease. Int J Cardiol 2001; 78(1):13-7.

S2-14. Kou MC, Wang L, Liang KJ, Wu M. Genotyping 5,10-methylenetetrahydrofolate reductase for patients with coronary artery disease in southern Taiwan. Journal of Food and Drug Analysis 9:173-7.

S2-15. Mager A, Lalezari S, Shohat T *et al*. Methylenetetrahydrofolate reductase genotypes and early-onset coronary artery disease. Circulation 1999; 100(24):2406-10.

S2-16. Morita H, Taguchi J, Kurihara H *et al*. Genetic polymorphism of 5,10-methylenetetrahydrofolate reductase (MTHFR) as a risk factor for coronary artery disease. Circulation 1997; 95(8):2032-6.

S2-17. Nakai K, Fusazaki T, Suzuki T *et al*. Genetic polymorphism of 5,10-methylenetetrahydrofolate increases risk of myocardial infarction and is correlated to elevated levels of homocysteine in the Japanese general population. Coron Artery Dis 2000; 11(1):47-51.

S2-18. Ou T, Yamakawa-Kobayashi K, Arinami T *et al*. Methylenetetrahydrofolate reductase and

apolipoprotein E polymorphisms are independent risk factors for coronary heart disease in Japanese: a case-control study. Atherosclerosis 1998; 137(1):23-8.

S2-19. Shioji K, Kokubo Y, Goto Y, Nonogi H, Iwai N. An association analysis between genetic polymorphisms of matrix metalloproteinase-3 and methylenetetrahydrofolate reductase and myocardial infarction in Japanese. J Thromb Haemost 2004; 2(3):527-8.

S2-20. Tokgozoglu SL, Alikasifoglu M, Unsal *et al*. Methylene tetrahydrofolate reductase genotype and the risk and extent of coronary artery disease in a population with low plasma folate. Heart 1999; 81(5):518-22.

S2-21. Yamada Y, Matsuo H, Segawa T *et al*. Assessment of genetic risk for myocardial infarction. Thromb Haemost 2006; 96(2):220-7.

S2-22. Zhang G, Dai C. Gene polymorphisms of homocysteine metabolism-related enzymes in Chinese patients with occlusive coronary artery or cerebral vascular diseases. Thromb Res 2001; 104(3):187-95.

S2-23. Zheng YZ, Tong J, Do XP, Pu XQ, Zhou BT. Prevalence of methylenetetrahydrofolate reductase C677T and its association with arterial and venous thrombosis in the Chinese population. Br J Haematol 2000; 109(4):870-4.

S2-24. Abbate R, Sardi I, Pepe G *et al*. The high prevalence of thermolabile 5-10 methylenetetrahydrofolate reductase (MTHFR) in Italians is not associated to an increased risk for coronary artery disease (CAD). Thromb Haemost 1998; 79(4):727-30.

S2-25. Ardissino D, Mannucci PM, Merlini PA *et al*. Prothrombotic genetic risk factors in young survivors of myocardial infarction. Blood 1999; 94(1):46-51.

S2-26. Fowkes FG, Lee AJ, Hau CM, Cooke A, Connor JM, Lowe GD. Methylene tetrahydrofolate reductase (MTHFR) and nitric oxide synthase (ecNOS) genes and risks of peripheral arterial disease and coronary heart disease: Edinburgh Artery Study. Atherosclerosis 2000; 150(1):179-85.

S2-27. Frederiksen J, Juul K, Grande P *et al*. Methylenetetrahydrofolate reductase polymorphism (C677T), hyperhomocysteinemia, and risk of ischemic cardiovascular disease and venous thromboembolism: prospective and case-control studies from the Copenhagen City Heart Study. Blood 2004; 104(10):3046-51.

S2-28. Gallagher PM, Meleady R, Shields DC *et al*. Homocysteine and risk of premature coronary heart disease. Evidence for a common gene mutation. Circulation 1996; 94(9):2154-8.

S2-29. Kluijtmans LA, Kastelein JJ, Lindemans J *et al*. Thermolabile methylenetetrahydrofolate reductase in coronary artery disease. Circulation 1997; 96(8):2573-7.

S2-30. Kostulas K, Crisby M, Huang WX *et al*. A methylenetetrahydrofolate reductase gene polymorphism in ischaemic stroke and in carotid artery stenosis. Eur J Clin Invest 1998; 28(4):285-9.

S2-31. Meleady R, Ueland PM, Blom H *et al*. Thermolabile methylenetetrahydrofolate reductase, homocysteine, and cardiovascular disease risk: the European Concerted Action Project. Am J Clin Nutr 2003; 77(1):63-70.

S2-32. Roest M, van der Schouw YT, Grobbee DE *et al*. Methylenetetrahydrofolate reductase 677 C/T genotype and cardiovascular disease mortality in postmenopausal women. Am J Epidemiol 2001; 153(7):673-9.

S2-33. Spiridonova MG, Stepanov VA, Pyzyrev VP, Karpov RS. [Relationship between polymorphism C677T of the methylene tetrahydrofolate reductase gene with clinical symptoms of coronary atherosclerosis]. Genetika 2000; 36(9):1269-73.

S2-34. Tanis BC, Blom HJ, Bloemenkamp DG *et al*. Folate, homocysteine levels, methylenetetrahydrofolate reductase (MTHFR) 677C --> T variant, and the risk of myocardial infarction in young women: effect of female hormones on homocysteine levels. J Thromb Haemost 2004; 2(1):35-41.

S2-35. Thogersen AM, Nilsson TK, Dahlen G *et al*. Homozygosity for the C677-->T mutation of

5,10-methylenetetrahydrofolate reductase and total plasma homocyst(e) ine are not associated with greater than normal risk of a first myocardial infarction in northern Sweden. Coron Artery Dis 2001; 12(2):85-90.

S2-36. Verhoef P, Kok FJ, Kluijtmans LA *et al*. The 677C-->T mutation in the methylenetetrahydrofolate reductase gene: associations with plasma total homocysteine levels and risk of coronary atherosclerotic disease. Atherosclerosis 1997; 132(1):105-13.

S2-37. Verhoeff BJ, Trip MD, Prins MH, Kastelein JJ, Reitsma PH. The effect of a common methylenetetrahydrofolate reductase mutation on levels of homocysteine, folate, vitamin B12 and on the risk of premature atherosclerosis. Atherosclerosis 1998; 141(1):161-6.

S2-38. Adams M, Smith PD, Martin D, Thompson JR, Lodwick D, Samani NJ. Genetic analysis of thermolabile methylenetetrahydrofolate reductase as a risk factor for myocardial infarction. QJM 1996; 89(6):437-44.

S2-39. Araujo F, Lopes M, Goncalves L, Maciel MJ, Cunha-Ribeiro LM. Hyperhomocysteinemia, MTHFR C677T genotype and low folate levels: a risk combination for acute coronary disease in a Portuguese population. Thromb Haemost 2000; 83(3):517-8.

S2-40. Benes P, Kankova K, Muzik J *et al*. Methylenetetrahydrofolate reductase polymorphism, type II diabetes mellitus, coronary artery disease, and essential hypertension in the Czech population. Mol Genet Metab 2001; 73(2):188-95.

S2-41. Brulhart MC, Dussoix P, Ruiz J, Passa P, Froguel P, James RW. The (Ala-Val) mutation of methylenetetrahydrofolate reductase as a genetic risk factor for vascular disease in non-insulin-dependent diabetic patients. Am J Hum Genet 1997; 60(1):228-9.

S2-42. Chambers JC, Ireland H, Thompson E *et al*. Methylenetetrahydrofolate reductase 677 C-->T mutation and coronary heart disease risk in UK Indian Asians. Arterioscler Thromb Vasc Biol 2000; 20(11):2448-52.

S2-43. Fernandez-Arcas N, Dieguez-Lucena JL, Munoz-Moran E *et al*. The genotype interactions of methylenetetrahydrofolate reductase and renin-angiotensin system genes are associated with myocardial infarction. Atherosclerosis 1999; 145(2):293-300.

S2-44. Ferrer . Unpublished individual participant data.

S2-45. Ferrer-Antunes C, Palmeiro A, Morais J, Lourenco M, Freitas M, Providencia L. The mutation C677T in the methylene tetrahydrofolate reductase gene as a risk factor for myocardial infarction in the Portuguese population. Thromb Haemost 1998; 80(3):521-2.

S2-46. Gardemann A, Weidemann H, Philipp M *et al*. The TT genotype of the methylenetetrahydrofolate reductase C677T gene polymorphism is associated with the extent of coronary atherosclerosis in patients at high risk for coronary artery disease. Eur Heart J 1999; 20(8):584-92.

S2-47. Gemmati D, Serino ML, Trivellato C, Fiorini S, Scapoli GL. C677T substitution in the methylenetetrahydrofolate reductase gene as a risk factor for venous thrombosis and arterial disease in selected patients. Haematologica 1999; 84(9):824-8.

S2-48. Girelli D, Martinelli N, Pizzolo F *et al*. The interaction between MTHFR 677 C-->T genotype and folate status is a determinant of coronary atherosclerosis risk. J Nutr 2003; 133(5):1281-5.

S2-49. Gorqcy I, Gorcqy J, Suliga M, Ciechanowicz A. C677T gene polymorphism of methelenetetrahydrofolate reductase (MTHFR) in patients with myocardial infarction. Polskie Archivum Medycyny Wewnetrzaej 1999; 4(10):849-54.

S2-50. Kadziela J, Janas J, Dzielinska Z *et al*. The C677T mutation in methylenetetrahydrofolate reductase gene, plasma homocysteine concentration and the risk of coronary artery disease. Kardiol Pol 2003; 59(7):17-26; discussion 26.

S2-51. Kolling K, Ndrepepa G, Koch W *et al*. Methylenetetrahydrofolate reductase gene C677T and A1298C polymorphisms, plasma homocysteine, folate, and vitamin B12 levels and the extent of coronary artery disease. Am J Cardiol 2004; 93(10):1201-6.

S2-52. Kozich. Unpublished individual participant data.

S2-53. Malik NM, Syrris P, Schwartzman R *et al*. Methylenetetrahydrofolate reductase polymorphism (C-677T) and coronary artery disease. Clin Sci (Lond) 1998; 95(3):311-5.

S2-54. Meisel C, Cascorbi I, Gerloff T *et al*. Identification of six methylenetetrahydrofolate reductase (MTHFR) genotypes resulting from common polymorphisms: impact on plasma homocysteine levels and development of coronary artery disease. Atherosclerosis 2001; 154(3):651-8.

S2-55. Pinto X, Vilaseca MA, Garcia-Giralt N *et al*. Homocysteine and the MTHFR 677C-->T allele in premature coronary artery disease. Case control and family studies. Eur J Clin Invest 2001; 31(1):24-30.

S2-56. Raslova K, Smolkova B, Vohnout B, Gasparovic J, Frohlich JJ. Risk factors for atherosclerosis in survivors of myocardial infarction and their spouses: comparison to controls without personal and family history of atherosclerosis. Metabolism 2001; 50(1):24-9.

S2-57. Reinhardt D, Sigusch HH, Vogt SF, Farker K, Muller S, Hoffmann A. Absence of association between a common mutation in the methylenetetrahydrofolate reductase gene and the risk of coronary artery disease. Eur J Clin Invest 1998; 28(1):20-3.

S2-58. Rossi GP, Maiolino G, Seccia TM *et al*. Hyperhomocysteinemia predicts total and cardiovascular mortality in high-risk women. J Hypertens 2006; 24(5):851-9.

S2-59. Rothenbacher D, Fischer HG, Hoffmeister A *et al*. Homocysteine and methylenetetrahydrofolate reductase genotype: association with risk of coronary heart disease and relation to inflammatory, hemostatic, and lipid parameters. Atherosclerosis 2002; 162(1):193-200.

S2-60. Sarecka-Hujar B, Zak I, Krauze J. Carrier-state of two or three polymorphic variants of MTHFR, IL-6 and ICAM1 genes increases the risk of coronary artery disease. Kardiol Pol 2008; 66(12):1269-77.

S2-61. Szczeklik A, Sanak M, Jankowski M *et al*. Mutation A1298C of methylenetetrahydrofolate reductase: risk for early coronary disease not associated with hyperhomocysteinemia. Am J Med Genet 2001; 101(1):36-9.

S2-62. Tobin MD, Braund PS, Burton PR *et al*. Genotypes and haplotypes predisposing to myocardial infarction: a multilocus case-control study. Eur Heart J 2004; 25(6):459-67.

S2-63. Todesco L, Angst C, Litynski P, Loehrer F, Fowler B, Haefeli WE. Methylenetetrahydrofolate reductase polymorphism, plasma homocysteine and age. Eur J Clin Invest 1999; 29(12):1003-9.

S2-64. Zuntar I, Topic E, Vukosavic D *et al*. Croatian population data for the C677T polymorphism in methylenetetrahydrofolate reductase: frequencies in healthy and atherosclerotic study groups. Clin Chim Acta 2003; 335(1-2):95-100.

S2-65. Brugada R, Marian AJ. A common mutation in methylenetetrahydrofolate reductase gene is not a major risk of coronary artery disease or myocardial infarction. Atherosclerosis 1997; 128(1):107-12.

S2-66. Dilley A, Hooper WC, El-Jamil M, Renshaw M, Wenger NK, Evatt BL. Mutations in the genes regulating methylene tetrahydrofolate reductase (MTHFR C-->T677) and cystathione beta-synthase (CBS G-->A919, CBS T-->c833) are not associated with myocardial infarction in African Americans. Thromb Res 2001; 103(2):109-15.

S2-67. Folsom AR, Nieto FJ, McGovern PG *et al*. Prospective study of coronary heart disease incidence in relation to fasting total homocysteine, related genetic polymorphisms, and B vitamins: the Atherosclerosis Risk in Communities (ARIC) study. Circulation 1998; 98(3):204-10.

S2-68. Hopkins PN, Wu LL, Wu J *et al*. Higher plasma homocyst(e)ine and increased susceptibility to adverse effects of low folate in early familial coronary artery disease. Arterioscler Thromb Vasc Biol 1995; 15(9):1314-20.

S2-69. Ma J, Stampfer MJ, Hennekens CH *et al*. Methylenetetrahydrofolate reductase polymorphism, plasma folate, homocysteine, and risk of myocardial infarction in US physicians. Circulation 1996; 94(10):2410-6.

S2-70. Schmitz C, Lindpaintner K, Verhoef P, Gaziano JM, Buring J. Genetic polymorphism of methylenetetrahydrofolate reductase and myocardial infarction. A case-control study. Circulation 1996; 94(8):1812-4.

S2-71. Schwartz SM, Siscovick DS, Malinow MR *et al*. Myocardial infarction in young women in relation to plasma total homocysteine, folate, and a common variant in the methylenetetrahydrofolate reductase gene. Circulation 1997; 96(2):412-7.

S2-72. Silberberg J, Crooks R, Fryer J *et al*. Gender differences and other determinants of the rise in plasma homocysteine after L-methionine loading. Atherosclerosis 1997; 133(1):105-10.

S2-73. Verhoef P, Rimm EB, Hunter DJ *et al*. A common mutation in the methylenetetrahydrofolate reductase gene and risk of coronary heart disease: results among U.S. men. J Am Coll Cardiol 1998; 32(2):353-9.

S2-74. Wilcken DEL, Wang XL, Sim AS, McCredie RM. Distribution in Healthy and Coronary Populations of the Methylenetetrahydrofolate Reductase (MTHFR) C677T Mutation. Arterioscler Thromb Vasc Biol 1996; 16(7):878-82.

S2-75. Anderson JL, King GJ, Thomson MJ *et al*. A mutation in the methylenetetrahydrofolate reductase gene is not associated with increased risk for coronary artery disease or myocardial infarction. J Am Coll Cardiol 1997; 30(5):1206-11.

S2-76. Brilakis ES, Berger PB, Ballman KV, Rozen R. Methylenetetrahydrofolate reductase (MTHFR) 677C>T and methionine synthase reductase (MTRR) 66A>G polymorphisms: association with serum homocysteine and angiographic coronary artery disease in the era of flour products fortified with folic acid. Atherosclerosis 2003; 168(2):315-22.

S2-77. Christensen B, Frosst P, Lussier-Cacan S *et al*. Correlation of a common mutation in the methylenetetrahydrofolate reductase gene with plasma homocysteine in patients with premature coronary artery disease. Arterioscler Thromb Vasc Biol 1997; 17(3):569-73.

S2-78. Hanson NQ, Aras O, Yang F, Tsai MY. C677T and A1298C polymorphisms of the methylenetetrahydrofolate reductase gene: incidence and effect of combined genotypes on plasma fasting and post-methionine load homocysteine in vascular disease. Clin Chem 2001; 47(4):661-6.

S2-79. Malinow MR, Nieto FJ, Kruger WD *et al*. The effects of folic acid supplementation on plasma total homocysteine are modulated by multivitamin use and methylenetetrahydrofolate reductase genotypes. Arterioscler Thromb Vasc Biol 1997; 17(6):1157-62.

S2-80. Payne DA, Chamoun AJ, Seifert SL, Stouffer GA. MTHFR 677 C-->T mutation: a predictor of early-onset coronary artery disease risk. Thromb Res 2001; 103(4):275-9.

S2-81. Topol EJ, McCarthy J, Gabriel S *et al*. Single nucleotide polymorphisms in multiple novel thrombospondin genes may be associated with familial premature myocardial infarction. Circulation 2001; 104(22):2641-4.

S2-82. Tsai MY, Welge BG, Hanson NQ *et al*. Genetic causes of mild hyperhomocysteinemia in patients with premature occlusive coronary artery diseases. Atherosclerosis 1999; 143(1):163-70.

S2-83. van Bockxmeer FM, Mamotte CD, Vasikaran SD, Taylor RR. Methylenetetrahydrofolate reductase gene and coronary artery disease. Circulation 1997; 95(1):21-3.

References for Table S3 (*MTHFR* and homocysteine levels)

S3-1. Chao CL, Tsai HH, Lee CM *et al*. The graded effect of hyperhomocysteinemia on the severity and extent of coronary atherosclerosis. Atherosclerosis 1999; 147(2):379-86.

S3-2. Nakai K, Fusazaki T, Suzuki T *et al*. Genetic polymorphism of 5,10-methylenetetrahydrofolate increases risk of myocardial infarction and is correlated to elevated levels of homocysteine in the Japanese general population. Coron Artery Dis 2000; 11(1):47-51.

S3-3. Ou T, Yamakawa-Kobayashi K, Arinami T *et al*. Methylenetetrahydrofolate reductase and apolipoprotein E polymorphisms are independent risk factors for coronary heart disease in Japanese: a case-control study. Atherosclerosis 1998; 137(1):23-8.

S3-4. Tokgozoglu SL, Alikasifoglu M, Unsal *et al*. Methylene tetrahydrofolate reductase genotype and the risk and extent of coronary artery disease in a population with low plasma folate. Heart 1999; 81(5):518-22.

S3-5. Friedman G, Goldschmidt N, Friedlander Y *et al*. A common mutation A1298C in human methylenetetrahydrofolate reductase gene: association with plasma total homocysteine and folate concentrations. J Nutr 1999; 129(9):1656-61.

S3-6. Ho CH. The influence of age, sex, vitamin B(12), folate levels and methylenetetrahydrofolate reductase C677T genetic mutations on plasma homocysteine in the Chinese population. Haematologica 2000; 85(10):1051-4.

S3-7. Jang Y, Park HY, Lee JH, Ryu HJ, Kim JY, Kim OY. A polymorphism of the methylenetetrahydrofolate reductase and methionine synthase gene in CAD patients: association with plasma folate, vitamin B12 and homocysteine. Nutrition Research 2002; 22(9):965-76.

S3-8. Kawashiri M, Kajinami K, Nohara A *et al*. Effect of common methylenetetrahydrofolate reductase gene mutation on coronary artery disease in familial hypercholesterolemia. Am J Cardiol 2000; 86(8):840-5.

S3-9. Kim CH, Hwang KY, Choi TM, Shin WY, Hong SY. The methylenetetrahydrofolate reductase gene polymorphism in Koreans with coronary artery disease. Int J Cardiol 2001; 78(1):13-7.

S3-10. Saw SM, Yuan JM, Ong CN *et al*. Genetic, dietary, and other lifestyle determinants of plasma homocysteine concentrations in middle-aged and older Chinese men and women in Singapore. Am J Clin Nutr 2001; 73(2):232-9.

S3-11. Somekawa Y, Kobayashi K, Tomura S, Aso T, Hamaguchi H. Effects of hormone replacement therapy and methylenetetrahydrofolate reductase polymorphism on plasma folate and homocysteine levels in postmenopausal Japanese women. Fertil Steril 2002; 77(3):481-6.

S3-12. Inamoto N, Katsuya T, Kokubo Y *et al*. Association of methylenetetrahydrofolate reductase gene polymorphism with carotid atherosclerosis depending on smoking status in a Japanese general population. Stroke 2003; 34(7):1628-33.

S3-13. Jee SH, Song KS, Shim WH *et al*. Major gene evidence after MTHFR-segregation analysis of serum homocysteine in families of patients undergoing coronary arteriography. Hum Genet 2002; 111(2):128-35.

S3-14. Yingdong Z, Zhigang Z, Yang L. Association of plasma homocysteine level and N5,N10-methylenetetrahydrofolate reductase gene polymorphism with cerebral infarction. Chin Med Sci J 2002; 17(4):231-5.

S3-15. Ilhan N, Kucuksu M, Kaman D, Ilhan N, Ozbay Y. The 677 C/T MTHFR polymorphism is associated with essential hypertension, coronary artery disease, and higher homocysteine levels. Arch Med Res 2008; 39(1):125-30.

S3-16. Thogersen AM, Nilsson TK, Dahlen G *et al*. Homozygosity for the C677-->T mutation of 5,10-methylenetetrahydrofolate reductase and total plasma homocyst(e) ine are not associated with greater than normal risk of a first myocardial infarction in northern Sweden. Coron Artery Dis 2001; 12(2):85-90.

S3-17. Kluijtmans LA, Kastelein JJ, Lindemans J *et al*. Thermolabile methylenetetrahydrofolate reductase in coronary artery disease. Circulation 1997; 96(8):2573-7.

S3-18. Meleady R, Ueland PM, Blom H *et al*. Thermolabile methylenetetrahydrofolate reductase, homocysteine, and cardiovascular disease risk: the European Concerted Action Project. Am J Clin Nutr 2003; 77(1):63-70.

S3-19. Devlin AM, Clarke R, Birks J, Evans JG, Halsted CH. Interactions among polymorphisms in folate-metabolizing genes and serum total homocysteine concentrations in a healthy elderly population. Am J Clin Nutr 2006; 83(3):708-13.

S3-20. Verhoef P, Kok FJ, Kluijtmans LA *et al*. The 677C-->T mutation in the methylenetetrahydrofolate reductase gene: associations with plasma total homocysteine levels and risk of coronary atherosclerotic disease. Atherosclerosis 1997; 132(1):105-13.

S3-21. Verhoeff BJ, Trip MD, Prins MH, Kastelein JJ, Reitsma PH. The effect of a common methylenetetrahydrofolate reductase mutation on levels of homocysteine, folate, vitamin B12 and on the risk of premature atherosclerosis. Atherosclerosis 1998; 141(1):161-6.

S3-22. Tanis BC, Blom HJ, Bloemenkamp DG *et al*. Folate, homocysteine levels, methylenetetrahydrofolate reductase (MTHFR) 677C --> T variant, and the risk of myocardial infarction in young women: effect of female hormones on homocysteine levels. J Thromb Haemost 2004; 2(1):35-41.

S3-23. Hustad S, Ueland PM, Vollset SE, Zhang Y, Bjorke-Monsen AL, Schneede J. Riboflavin as a determinant of plasma total homocysteine: effect modification by the methylenetetrahydrofolate reductase C677T polymorphism. Clin Chem 2000; 46(8 Pt 1):1065-71.

S3-24. Silaste ML, Rantala M, Sampi M, Alfthan G, Aro A, Kesaniemi YA. Polymorphisms of key enzymes in homocysteine metabolism affect diet responsiveness of plasma homocysteine in healthy women. J Nutr 2001; 131(10):2643-7.

S3-25. De Bree A, Verschuren WM, Kromhout D, Kluijtmans LA, Blom HJ. Homocysteine determinants and the evidence to what extent homocysteine determines the risk of coronary heart disease. Pharmacol Rev 2002; 54(4):599-618.

S3-26. Bathum L, Petersen I, Christiansen L, Konieczna A, Sorensen TI, Kyvik KO. Genetic and environmental influences on plasma homocysteine: results from a Danish twin study. Clin Chem 2007; 53(5):971-9.

S3-27. Frederiksen J, Juul K, Grande P *et al*. Methylenetetrahydrofolate reductase polymorphism (C677T), hyperhomocysteinemia, and risk of ischemic cardiovascular disease and venous thromboembolism: prospective and case-control studies from the Copenhagen City Heart Study. Blood 2004; 104(10):3046-51.

S3-28. Husemoen LL, Toft U, Fenger M, Jorgensen T, Johansen N, Linneberg A. The association between atopy and factors influencing folate metabolism: is low folate status causally related to the development of atopy? Int J Epidemiol 2006; 35(4):954-61.

S3-29. Hustad S, Midttun O, Schneede J, Vollset SE, Grotmol T, Ueland PM. The methylenetetrahydrofolate reductase 677C-->T polymorphism as a modulator of a B vitamin network with major effects on homocysteine metabolism. Am J Hum Genet 2007; 80(5):846-55.

S3-30. Dekou V, Whincup P, Papacosta O *et al*. The effect of the C677T and A1298C polymorphisms in the methylenetetrahydrofolate reductase gene on homocysteine levels in elderly men and women from the British regional heart study. Atherosclerosis 2001; 154(3):659-66.

S3-31. Meisel C, Cascorbi I, Gerloff T *et al*. Identification of six methylenetetrahydrofolate reductase (MTHFR) genotypes resulting from common polymorphisms: impact on plasma homocysteine levels and development of coronary artery disease. Atherosclerosis 2001; 154(3):651-8.

S3-32. Rothenbacher D, Fischer HG, Hoffmeister A *et al*. Homocysteine and methylenetetrahydrofolate reductase genotype: association with risk of coronary heart

disease and relation to inflammatory, hemostatic, and lipid parameters. Atherosclerosis 2002; 162(1):193-200.

S3-33. Chango A, Boisson F, Barbe F *et al*. The effect of 677C-->T and 1298A-->C mutations on plasma homocysteine and 5,10-methylenetetrahydrofolate reductase activity in healthy subjects. Br J Nutr 2000; 83(6):593-6.

S3-34. Chango A, Potier De Courcy G, Boisson F *et al*. 5,10-methylenetetrahydrofolate reductase common mutations, folate status and plasma homocysteine in healthy French adults of the Supplementation en Vitamines et Mineraux Antioxydants (SU.VI.MAX) cohort. Br J Nutr 2000; 84(6):891-6.

S3-35. Girelli D, Friso S, Trabetti E *et al*. Methylenetetrahydrofolate reductase C677T mutation, plasma homocysteine, and folate in subjects from northern Italy with or without angiographically documented severe coronary atherosclerotic disease: evidence for an important genetic-environmental interaction. Blood 1998; 91(11):4158-63.

S3-36. Ferrer . Unpublished individual participant data.

S3-37. Thuillier L, Chadefaux-Vekemans B, Bonnefont JP *et al*. Does the polymorphism 677C-T of the 5,10-methylenetetrahydrofolate reductase gene contribute to homocysteine-related vascular disease? J Inherit Metab Dis 1998; 21(8):812-22.

S3-38. Zittoun J, Tonetti C, Bories D, Pignon JM, Tulliez M. Plasma homocysteine levels related to interactions between folate status and methylenetetrahydrofolate reductase: a study in 52 healthy subjects. Metabolism 1998; 47(11):1413-8.

S3-39. Chambers JC, Ireland H, Thompson E *et al*. Methylenetetrahydrofolate reductase 677 C-->T mutation and coronary heart disease risk in UK Indian Asians. Arterioscler Thromb Vasc Biol 2000; 20(11):2448-52.

S3-40. Gemmati D, Serino ML, Trivellato C, Fiorini S, Scapoli GL. C677T substitution in the methylenetetrahydrofolate reductase gene as a risk factor for venous thrombosis and arterial disease in selected patients. Haematologica 1999; 84(9):824-8.

S3-41. Kozich. Unpublished individual participant data.

S3-42. Todesco L, Angst C, Litynski P, Loehrer F, Fowler B, Haefeli WE. Methylenetetrahydrofolate reductase polymorphism, plasma homocysteine and age. Eur J Clin Invest 1999; 29(12):1003-9.

S3-43. D'Angelo A, Coppola A, Madonna P *et al*. The role of vitamin B12 in fasting hyperhomocysteinemia and its interaction with the homozygous C677T mutation of the methylenetetrahydrofolate reductase (MTHFR) gene. A case-control study of patients with early-onset thrombotic events. Thromb Haemost 2000; 83(4):563-70.

S3-44. Passaro A, Vanini A, Calzoni F *et al*. Plasma homocysteine, methylenetetrahydrofolate reductase mutation and carotid damage in elderly healthy women. Atherosclerosis 2001; 157(1):175-80.

S3-45. Szczeklik A, Sanak M, Jankowski M *et al*. Mutation A1298C of methylenetetrahydrofolate reductase: risk for early coronary disease not associated with hyperhomocysteinemia. Am J Med Genet 2001; 101(1):36-9.

S3-46. Cappuccio FP, Bell R, Perry IJ *et al*. Homocysteine levels in men and women of different ethnic and cultural background living in England. Atherosclerosis 2002; 164(1):95-102.

S3-47. Fohr IP, Prinz-Langenohl R, Bronstrup A *et al*. 5,10-Methylenetetrahydrofolate reductase genotype determines the plasma homocysteine-lowering effect of supplementation with 5-methyltetrahydrofolate or folic acid in healthy young women. Am J Clin Nutr 2002; 75(2):275-82.

S3-48. Madonna P, de Stefano V, Coppola A *et al*. Hyperhomocysteinemia and other inherited prothrombotic conditions in young adults with a history of ischemic stroke. Stroke 2002; 33(1):51-6.

S3-49. Pullin CH, Ashfield-Watt PA, Burr ML *et al*. Optimization of dietary folate or low-dose folic

acid supplements lower homocysteine but do not enhance endothelial function in healthy adults, irrespective of the methylenetetrahydrofolate reductase (C677T) genotype. J Am Coll Cardiol 2001; 38(7):1799-805.

S3-50. Castro R, Rivera I, Ravasco P *et al*. 5,10-Methylenetetrahydrofolate reductase 677C-->T and 1298A-->C mutations are genetic determinants of elevated homocysteine. QJM 2003; 96(4):297-303.

S3-51. Litynski P, Loehrer F, Linder L, Todesco L, Fowler B. Effect of low doses of 5-methyltetrahydrofolate and folic acid on plasma homocysteine in healthy subjects with or without the 677C-->T polymorphism of methylenetetrahydrofolate reductase. Eur J Clin Invest 2002; 32(9):662-8.

S3-52. Reyes-Engel A, Munoz E, Gaitan MJ *et al*. Implications on human fertility of the 677C-->T and 1298A-->C polymorphisms of the MTHFR gene: consequences of a possible genetic selection. Mol Hum Reprod 2002; 8(10):952-7.

S3-53. Kolling K, Ndrepepa G, Koch W *et al*. Methylenetetrahydrofolate reductase gene C677T and A1298C polymorphisms, plasma homocysteine, folate, and vitamin B12 levels and the extent of coronary artery disease. Am J Cardiol 2004; 93(10):1201-6.

S3-54. Ma J, Stampfer MJ, Hennekens CH *et al*. Methylenetetrahydrofolate reductase polymorphism, plasma folate, homocysteine, and risk of myocardial infarction in US physicians. Circulation 1996; 94(10):2410-6.

S3-55. Schmitz C, Lindpaintner K, Verhoef P, Gaziano JM, Buring J. Genetic polymorphism of methylenetetrahydrofolate reductase and myocardial infarction. A case-control study. Circulation 1996; 94(8):1812-4.

S3-56. Folsom AR, Nieto FJ, McGovern PG *et al*. Prospective study of coronary heart disease incidence in relation to fasting total homocysteine, related genetic polymorphisms, and B vitamins: the Atherosclerosis Risk in Communities (ARIC) study. Circulation 1998; 98(3):204-10.

S3-57. Jacques PF, Kalmbach R, Bagley PJ *et al*. The relationship between riboflavin and plasma total homocysteine in the Framingham Offspring cohort is influenced by folate status and the C677T transition in the methylenetetrahydrofolate reductase gene. J Nutr 2002; 132(2):283-8.

S3-58. Schwartz SM, Siscovick DS, Malinow MR *et al*. Myocardial infarction in young women in relation to plasma total homocysteine, folate, and a common variant in the methylenetetrahydrofolate reductase gene. Circulation 1997; 96(2):412-7.

S3-59. Silberberg J, Crooks R, Fryer J *et al*. Gender differences and other determinants of the rise in plasma homocysteine after L-methionine loading. Atherosclerosis 1997; 133(1):105-10.

S3-60. Zee RY, Mora S, Cheng S *et al*. Homocysteine, 5,10-methylenetetrahydrofolate reductase 677C>T polymorphism, nutrient intake, and incident cardiovascular disease in 24,968 initially healthy women. Clin Chem 2007; 53(5):845-51.

S3-61. Hopkins PN, Wu LL, Wu J *et al*. Higher plasma homocyst(e)ine and increased susceptibility to adverse effects of low folate in early familial coronary artery disease. Arterioscler Thromb Vasc Biol 1995; 15(9):1314-20.

S3-62. Christensen B, Frosst P, Lussier-Cacan S *et al*. Correlation of a common mutation in the methylenetetrahydrofolate reductase gene with plasma homocysteine in patients with premature coronary artery disease. Arterioscler Thromb Vasc Biol 1997; 17(3):569-73.

S3-63. Malinow MR, Nieto FJ, Kruger WD *et al*. The effects of folic acid supplementation on plasma total homocysteine are modulated by multivitamin use and methylenetetrahydrofolate reductase genotypes. Arterioscler Thromb Vasc Biol 1997; 17(6):1157-62.

S3-64. Anderson JL, King GJ, Thomson MJ *et al*. A mutation in the methylenetetrahydrofolate reductase gene is not associated with increased risk for coronary artery disease or myocardial infarction. J Am Coll Cardiol 1997; 30(5):1206-11.

S3-65. Eikelboom JW, Hankey GJ, Anand SS, Lofthouse E, Staples N, Baker RI. Association

between high homocyst(e)ine and ischemic stroke due to large- and small-artery disease but not other etiologic subtypes of ischemic stroke. Stroke 2000; 31(5):1069-75.

S3-66. Tsai MY, Welge BG, Hanson NQ *et al*. Genetic causes of mild hyperhomocysteinemia in patients with premature occlusive coronary artery diseases. Atherosclerosis 1999; 143(1):163-70.

S3-67. Hanson NQ, Aras O, Yang F, Tsai MY. C677T and A1298C polymorphisms of the methylenetetrahydrofolate reductase gene: incidence and effect of combined genotypes on plasma fasting and post-methionine load homocysteine in vascular disease. Clin Chem 2001; 47(4):661-6.

S3-68. Caudill MA, Le T, Moonie SA, Esfahani ST, Cogger EA. Folate status in women of childbearing age residing in Southern California after folic acid fortification. J Am Coll Nutr 2001; 20(2 Suppl):129-34.

S3-69. Guinotte CL, Burns MG, Axume JA *et al*. Methylenetetrahydrofolate reductase 677C-->T variant modulates folate status response to controlled folate intakes in young women. J Nutr 2003; 133(5):1272-80.

References for Table S4 (Population surveys of folate levels)

e4-1. Ronnenberg AG, Goldman MB, Aitken IW, Xu X. Anemia and deficiencies of folate and vitamin B-6 are common and vary with season in Chinese women of childbearing age. J Nutr 2000; 130(11):2703-10.

S4-2. Wang YL., Zhou ZH, Wang MZ, Lu ZY, Ming YC. Study of Normal Level Serum Folate and Vitamin B12 Among Healthy Adults in Hubei Province . Journal of Mathematical Medicine 2000; 1(13):48-9.

S4-3. Friedman G, Goldschmidt N, Friedlander Y *et al*. A common mutation A1298C in human methylenetetrahydrofolate reductase gene: association with plasma total homocysteine and folate concentrations. J Nutr 1999; 129(9):1656-61.

S4-4. Jang Y, Park HY, Lee JH, Ryu HJ, Kim JY, Kim OY. A polymorphism of the methylenetetrahydrofolate reductase and methionine synthase gene in CAD patients: association with plasma folate, vitamin B12 and homocysteine. Nutrition Research 2002; 22(9):965-76.

S4-5. Saw SM, Yuan JM, Ong CN *et al*. Genetic, dietary, and other lifestyle determinants of plasma homocysteine concentrations in middle-aged and older Chinese men and women in Singapore. Am J Clin Nutr 2001; 73(2):232-9.

S4-6. Hao L, Ma J, Stampfer MJ *et al*. Geographical, seasonal and gender differences in folate status among Chinese adults. J Nutr 2003; 133(11):3630-5.

S4-7. Jee SH, Song KS, Shim WH *et al*. Major gene evidence after MTHFR-segregation analysis of serum homocysteine in families of patients undergoing coronary arteriography. Hum Genet 2002; 111(2):128-35.

S4-8. Hultdin J, Van Guelpen B, Bergh A, Hallmans G, Stattin P. Plasma folate, vitamin B12, and homocysteine and prostate cancer risk: a prospective study. Int J Cancer 2005; 113(5):819-24.

S4-9. Van Guelpen B, Hultdin J, Johansson I *et al*. Folate, Vitamin B12, and Risk of Ischemic and Hemorrhagic Stroke: A Prospective, Nested Case-Referent Study of Plasma Concentrations and Dietary Intake. Stroke 2005; 36(7):1426-31.

S4-10. Hartman TJ, Woodson K, Stolzenberg-Solomon R *et al*. Association of the B-vitamins pyridoxal 5'-phosphate (B(6)), B(12), and folate with lung cancer risk in older men. Am J Epidemiol 2001; 153(7):688-94.

S4-11. Meleady R, Ueland PM, Blom H *et al*. Thermolabile methylenetetrahydrofolate reductase, homocysteine, and cardiovascular disease risk: the European Concerted Action Project. Am J Clin Nutr 2003; 77(1):63-70.

S4-12. Nurk E, Tell GS, Vollset SE *et al*. Changes in lifestyle and plasma total homocysteine: the Hordaland Homocysteine Study. Am J Clin Nutr 2004; 79(5):812-9.

S4-13. Voutilainen S, Virtanen JK, Rissanen TH *et al*. Serum folate and homocysteine and the incidence of acute coronary events: the Kuopio Ischaemic Heart Disease Risk Factor Study. Am J Clin Nutr 2004; 80(2):317-23.

S4-14. Clarke R, Woodhouse P, Ulvik A *et al*. Variability and determinants of total homocysteine concentrations in plasma in an elderly population. Clin Chem 1998; 44(1):102-7.

S4-15. Devlin AM, Clarke R, Birks J, Evans JG, Halsted CH. Interactions among polymorphisms in folate-metabolizing genes and serum total homocysteine concentrations in a healthy elderly population. Am J Clin Nutr 2006; 83(3):708-13.

S4-16. de Bree A, Verschuren WM, Blom HJ, Kromhout D. Association between B vitamin intake and plasma homocysteine concentration in the general Dutch population aged 20-65 y. Am J Clin Nutr 2001; 73(6):1027-33.

S4-17. Dierkes J. Vitamin requirements for the reduction of homocysteine blood levels in healthy young women Bonn: Univeristy of Bonn, 1995.

S4-18. Melse-Boonstra A, de Bree A, Verhoef P, Bjorke-Monsen AL, Verschuren WM. Dietary monoglutamate and polyglutamate folate are associated with plasma folate concentrations in Dutch men and women aged 20-65 years. J Nutr 2002; 132(6):1307-12.

S4-19. Naurath HJ, Joosten E, Riezler R, Stabler SP, Allen RH, Lindenbaum J. Effects of vitamin B12, folate, and vitamin B6 supplements in elderly people with normal serum vitamin concentrations. Lancet 1995; 346(8967):85-9.

S4-20. Woodside JV, Yarnell JW, McMaster D *et al*. Effect of B-group vitamins and antioxidant vitamins on hyperhomocysteinemia: a double-blind, randomized, factorial-design, controlled trial. Am J Clin Nutr 1998; 67(5):858-66.

S4-21. Alfthan G, Laurinen MS, Valsta LM, Pastinen T, Aro A. Folate intake, plasma folate and homocysteine status in a random Finnish population. Eur J Clin Nutr 2003; 57(1):81-8.

S4-22. Wahlin A, Backman L, Hultdin J, Adolfsson R, Nilsson LG. Reference values for serum levels of vitamin B12 and folic acid in a population-based sample of adults between 35 and 80 years of age. Public Health Nutr 2002; 5(3):505-11.

S4-23. Brouwer IA, van Dusseldorp M, Duran M *et al*. Low-dose folic acid supplementation does not influence plasma methionine concentrations in young non-pregnant women. Br J Nutr 1999; 82(2):85-9.

S4-24. Tanis BC, Blom HJ, Bloemenkamp DG *et al*. Folate, homocysteine levels, methylenetetrahydrofolate reductase (MTHFR) 677C --> T variant, and the risk of myocardial infarction in young women: effect of female hormones on homocysteine levels. J Thromb Haemost 2004; 2(1):35-41.

S4-25. Durga J, van Boxtel MP, Schouten EG *et al*. Effect of 3-year folic acid supplementation on cognitive function in older adults in the FACIT trial: a randomised, double blind, controlled trial. Lancet 2007; 369(9557):208-16.

S4-26. Hustad S, Ueland PM, Vollset SE, Zhang Y, Bjorke-Monsen AL, Schneede J. Riboflavin as a determinant of plasma total homocysteine: effect modification by the methylenetetrahydrofolate reductase C677T polymorphism. Clin Chem 2000; 46(8 Pt 1):1065-71.

S4-27. Bjorkegren K, Svardsudd K. A population-based intervention study on elevated serum levels of methylmalonic acid and total homocysteine in elderly people: results after 36 months of follow-up. J Intern Med 2004; 256(5):446-52.

S4-28. Hustad S, Midttun O, Schneede J, Vollset SE, Grotmol T, Ueland PM. The methylenetetrahydrofolate reductase 677C-->T polymorphism as a modulator of a B vitamin network with major effects on homocysteine metabolism. Am J Hum Genet 2007; 80(5):846-55.

S4-29. Dekou V, Whincup P, Papacosta O *et al*. The effect of the C677T and A1298C polymorphisms in the methylenetetrahydrofolate reductase gene on homocysteine levels in elderly men and women from the British regional heart study. Atherosclerosis 2001; 154(3):659-66.

S4-30. Trobs M, Renner T, Scherer G *et al*. Nutrition, antioxidants, and risk factor profile of nonsmokers, passive smokers and smokers of the Prevention Education Program (PEP) in Nuremberg, Germany. Prev Med 2002; 34(6):600-7.

S4-31. Brown KS, Kluijtmans LA, Young IS *et al*. Genetic evidence that nitric oxide modulates homocysteine: the NOS3 894TT genotype is a risk factor for hyperhomocystenemia. Arterioscler Thromb Vasc Biol 2003; 23(6):1014-20.

S4-32. Chango A, Potier De Courcy G, Boisson F *et al*. 5,10-methylenetetrahydrofolate reductase common mutations, folate status and plasma homocysteine in healthy French adults of the Supplementation en Vitamines et Mineraux Antioxydants (SU.VI.MAX) cohort. Br J Nutr 2000; 84(6):891-6.

S4-33. Girelli D, Friso S, Trabetti E *et al*. Methylenetetrahydrofolate reductase C677T mutation, plasma homocysteine, and folate in subjects from northern Italy with or without angiographically documented severe coronary atherosclerotic disease: evidence for an

important genetic-environmental interaction. Blood 1998; 91(11):4158-63.

S4-34. Dierkes J, Kroesen M, Pietrzik K. Folic acid and Vitamin B6 supplementation and plasma homocysteine concentrations in healthy young women. Int J Vitam Nutr Res 1998; 68(2):98-103.

S4-35. Friso S, Girelli D, Martinelli N *et al*. Low plasma vitamin B-6 concentrations and modulation of coronary artery disease risk. Am J Clin Nutr 2004; 79(6):992-8.

S4-36. Chambers JC, Obeid OA, Refsum H *et al*. Plasma homocysteine concentrations and risk of coronary heart disease in UK Indian Asian and European men. Lancet 2000; 355(9203):523-7.

S4-37. Duthie SJ, Whalley LJ, Collins AR, Leaper S, Berger K, Deary IJ. Homocysteine, B vitamin status, and cognitive function in the elderly. Am J Clin Nutr 2002; 75(5):908-13.

S4-38. Gemmati D, Serino ML, Trivellato C, Fiorini S, Scapoli GL. C677T substitution in the methylenetetrahydrofolate reductase gene as a risk factor for venous thrombosis and arterial disease in selected patients. Haematologica 1999; 84(9):824-8.

S4-39. Kozich. Unpublished individual participant data.

S4-40. MacMahon M, Kirkpatrick C, Cummings CE *et al*. A pilot study with simvastatin and folic acid/vitamin B12 in preparation for the Study of the Effectiveness of Additional Reductions in Cholesterol and Homocysteine (SEARCH). Nutr Metab Cardiovasc Dis 2000; 10(4):195-203.

S4-41. Ravaglia G, Forti P, Maioli F *et al*. Homocysteine and folate as risk factors for dementia and Alzheimer disease. Am J Clin Nutr 2005; 82(3):636-43.

S4-42. Ashfield-Watt PA, Pullin CH, Whiting JM *et al*. Methylenetetrahydrofolate reductase 677C-->T genotype modulates homocysteine responses to a folate-rich diet or a low-dose folic acid supplement: a randomized controlled trial. Am J Clin Nutr 2002; 76(1):180-6.

S4-43. D'Angelo A, Coppola A, Madonna P *et al*. The role of vitamin B12 in fasting hyperhomocysteinemia and its interaction with the homozygous C677T mutation of the methylenetetrahydrofolate reductase (MTHFR) gene. A case-control study of patients with early-onset thrombotic events. Thromb Haemost 2000; 83(4):563-70.

S4-44. Dierkes J, Jeckel A, Ambrosch A, Westphal S, Luley C, Boeing H. Factors explaining the difference of total homocysteine between men and women in the European Investigation Into Cancer and Nutrition Potsdam study. Metabolism 2001; 50(6):640-5.

S4-45. Mayer O Jr, Simon J, Rosolova H. A population study of the influence of beer consumption on folate and homocysteine concentrations. Eur J Clin Nutr 2001; 55(7):605-9.

S4-46. Rauh M, Verwied S, Knerr I, Dorr HG, Sonnichsen A, Koletzko B. Homocysteine concentrations in a German cohort of 500 individuals: reference ranges and determinants of plasma levels in healthy children and their parents. Amino Acids 2001; 20(4):409-18.

S4-47. Szczeklik A, Sanak M, Jankowski M *et al*. Mutation A1298C of methylenetetrahydrofolate reductase: risk for early coronary disease not associated with hyperhomocysteinemia. Am J Med Genet 2001; 101(1):36-9.

S4-48. Fohr IP, Prinz-Langenohl R, Bronstrup A *et al*. 5,10-Methylenetetrahydrofolate reductase genotype determines the plasma homocysteine-lowering effect of supplementation with 5-methyltetrahydrofolate or folic acid in healthy young women. Am J Clin Nutr 2002; 75(2):275-82.

S4-49. Pullin CH, Ashfield-Watt PA, Burr ML *et al*. Optimization of dietary folate or low-dose folic acid supplements lower homocysteine but do not enhance endothelial function in healthy adults, irrespective of the methylenetetrahydrofolate reductase (C677T) genotype. J Am Coll Cardiol 2001; 38(7):1799-805.

S4-50. Ruston D, Hoare J, Henderson L *et al*. Nutritional Status (Anthropometry and Blood Analytes) Blood Pressure and Physical Activitiy . The National Diet and Nutrition Survey: Adults Aged 19 to 64 years. London: The Stationary Office, 2004; vol 4).

S4-51. Wolters M, Hermann S, Hahn A. B vitamin status and concentrations of homocysteine and methylmalonic acid in elderly German women. Am J Clin Nutr 2003; 78(4):765-72.

S4-52. Castro R, Rivera I, Ravasco P *et al*. 5,10-Methylenetetrahydrofolate reductase 677C-->T and 1298A-->C mutations are genetic determinants of elevated homocysteine. QJM 2003; 96(4):297-303.

S4-53. Hatzis CM, Bertsias GK, Linardakis M, Scott JM, Kafatos AG. Dietary and other lifestyle correlates of serum folate concentrations in a healthy adult population in Crete, Greece: a cross-sectional study. Nutr J 2006; 5:5.

S4-54. Kadziela J, Janas J, Dzielinska Z *et al*. The C677T mutation in methylenetetrahydrofolate reductase gene, plasma homocysteine concentration and the risk of coronary artery disease. Kardiol Pol 2003; 59(7):17-26; discussion 26.

S4-55. Kolling K, Ndrepepa G, Koch W *et al*. Methylenetetrahydrofolate reductase gene C677T and A1298C polymorphisms, plasma homocysteine, folate, and vitamin B12 levels and the extent of coronary artery disease. Am J Cardiol 2004; 93(10):1201-6.

S4-56. Nelson M, Erens B, Bates B, Church S, Boshier T. Nutritional status, physical activity, economic, social and other factors. The National Diet and Nutrition Survey: Adults aged 19 to 64 years. London: The Stationary Office, 2004; vol 3).

S4-57. Obeid R, Schorr H, Eckert R, Herrmann W. Vitamin B12 status in the elderly as judged by available biochemical markers. Clin Chem 2004; 50(1):238-41.

S4-58. Ma J, Stampfer MJ, Hennekens CH *et al*. Methylenetetrahydrofolate reductase polymorphism, plasma folate, homocysteine, and risk of myocardial infarction in US physicians. Circulation 1996; 94(10):2410-6.

S4-59. Verhoef P, Rimm EB, Hunter DJ *et al*. A common mutation in the methylenetetrahydrofolate reductase gene and risk of coronary heart disease: results among U.S. men. J Am Coll Cardiol 1998; 32(2):353-9.

S4-60. Folsom AR, Nieto FJ, McGovern PG *et al*. Prospective study of coronary heart disease incidence in relation to fasting total homocysteine, related genetic polymorphisms, and B vitamins: the Atherosclerosis Risk in Communities (ARIC) study. Circulation 1998; 98(3):204-10.

S4-61. Pfeiffer CM, Johnson CL, Jain RB *et al*. Trends in blood folate and vitamin B-12 concentrations in the United States, 1988 2004. Am J Clin Nutr 2007; 86(3):718-27.

S4-62. Zhang SM, Willett WC, Selhub J *et al*. Plasma folate, vitamin B6, vitamin B12, homocysteine, and risk of breast cancer. J Natl Cancer Inst 2003; 95(5):373-80.

S4-63. Dalery K, Lussier-Cacan S, Selhub J, Davignon J, Latour Y, Genest J Jr. Homocysteine and coronary artery disease in French Canadian subjects: relation with vitamins B12, B6, pyridoxal phosphate, and folate. Am J Cardiol 1995; 75(16):1107-11.

S4-64. Jacques PF, Selhub J, Bostom AG, Wilson PW, Rosenberg IH. The effect of folic acid fortification on plasma folate and total homocysteine concentrations. N Engl J Med 1999; 340(19):1449-54.

S4-65. Jacques PF, Kalmbach R, Bagley PJ *et al*. The relationship between riboflavin and plasma total homocysteine in the Framingham Offspring cohort is influenced by folate status and the C677T transition in the methylenetetrahydrofolate reductase gene. J Nutr 2002; 132(2):283-8.

S4-66. Schwartz SM, Siscovick DS, Malinow MR *et al*. Myocardial infarction in young women in relation to plasma total homocysteine, folate, and a common variant in the methylenetetrahydrofolate reductase gene. Circulation 1997; 96(2):412-7.

S4-67. Hopkins PN, Wu LL, Wu J *et al*. Higher plasma homocyst(e)ine and increased susceptibility to adverse effects of low folate in early familial coronary artery disease. Arterioscler Thromb Vasc Biol 1995; 15(9):1314-20.

S4-68. Jacques PF, Bostom AG, Williams RR *et al*. Relation between folate status, a common

mutation in methylenetetrahydrofolate reductase, and plasma homocysteine concentrations. Circulation 1996; 93(1):7-9.

S4-69. Lawrence JM, Petitti DB, Watkins M, Umekubo MA. Trends in serum folate after food fortification. Lancet 1999; 354(9182):915-6.

S4-70. Robinson K, Mayer EL, Miller DP *et al*. Hyperhomocysteinemia and low pyridoxal phosphate. Common and independent reversible risk factors for coronary artery disease. Circulation 1995; 92(10):2825-30.

S4-71. Malinow MR, Nieto FJ, Kruger WD *et al*. The Effects of Folic Acid Supplementation on Plasma Total Homocysteine Are Modulated by Multivitamin Use and Methylenetetrahydrofolate Reductase Genotypes. Arterioscler Thromb Vasc Biol 1997; 17(6):1157-62.

S4-72. Aronow WS, Ahn C, Schoenfeld MR. Association between plasma homocysteine and extracranial carotid arterial disease in older persons. Am J Cardiol 1997; 79(10):1432-3.

S4-73. Eikelboom JW, Hankey GJ, Anand SS, Lofthouse E, Staples N, Baker RI. Association between high homocyst(e)ine and ischemic stroke due to large- and small-artery disease but not other etiologic subtypes of ischemic stroke. Stroke 2000; 31(5):1069-75.

S4-74. Caudill MA, Le T, Moonie SA, Esfahani ST, Cogger EA. Folate status in women of childbearing age residing in Southern California after folic acid fortification. J Am Coll Nutr 2001; 20(2 Suppl):129-34.

S4-75. Hickling S, Hung J, Knuiman M *et al*. Impact of voluntary folate fortification on plasma homocysteine and serum folate in Australia from 1995 to 2001: a population based cohort study. J Epidemiol Community Health 2005; 59(5):371-6.

S4-76. McMahon JA, Green TJ, Skeaff CM, Knight RG, Mann JI, Williams SM. A controlled trial of homocysteine lowering and cognitive performance. N Engl J Med 2006; 354(26):2764-72.

References for Table S5 (Randomised trials that assessed the effects of folic acid on major coronary events)

S5-1. Galan P, Kesse-Guyot E, Czernichow S, Briancon S, Blacher J, Hercberg S. Effects of B vitamins and omega 3 fatty acids on cardiovascular diseases: a randomised placebo controlled trial. BMJ 2010; 341:c6273.

S5-2. Clarke R, Halsey J, Lewington S *et al*. Effects of lowering homocysteine levels with B vitamins on cardiovascular disease, cancer, and cause-specific mortality: Meta-analysis of 8 randomized trials involving 37 485 individuals. Arch Intern Med 2010; 170(18):1622-31.

S5-3. VITATOPS Collaborative Group. B vitamins in patients with recent transient ischaemic attack or stroke in the VITAmins TO Prevent Stroke (VITATOPS) trial: a randomised, double-blind, parallel, placebo-controlled trial. Lancet Neurol 2010; 9(9):855-65.

S5-4. Heartwire. No CV risk reduction with B vitamins posttransplant [Web Page]. Available at http://www.theheart.org/article/1018889.do. (Accessed 6 April 2011).
